# Comparing the Chemistry of Malvidin-3-*O*-glucoside and Malvidin-3,5-*O*-diglucoside Networks: A Holistic Approach to the Acidic and Basic Paradigms with Implications in Biological Studies

**DOI:** 10.1021/acs.jafc.4c00552

**Published:** 2024-03-23

**Authors:** André Seco, Ana Rita Pereira, Ambrósio Camuenho, Joana Oliveira, Ricardo Dias, Natércia F. Brás, Nuno Basílio, A. Jorge Parola, João C. Lima, Victor de Freitas, Fernando Pina

**Affiliations:** † LAQV—REQUIMTE, Departamento de Química, Faculdade de Ciências e Tecnologia, Universidade Nova de Lisboa, 2829-516 Caparica, Portugal; ‡ LAQV—REQUIMTE, Departamento de Química e Bioquímica, Faculdade de Ciências, Universidade do Porto, Rua do Campo Alegre, 687, 4169-007 Porto, Portugal

**Keywords:** anthocyanins, malvidin monoglucoside and diglucoside, degradation rates, stopped-flow, kinetics, malvidin monoglucoside dimers, physiologic pH

## Abstract

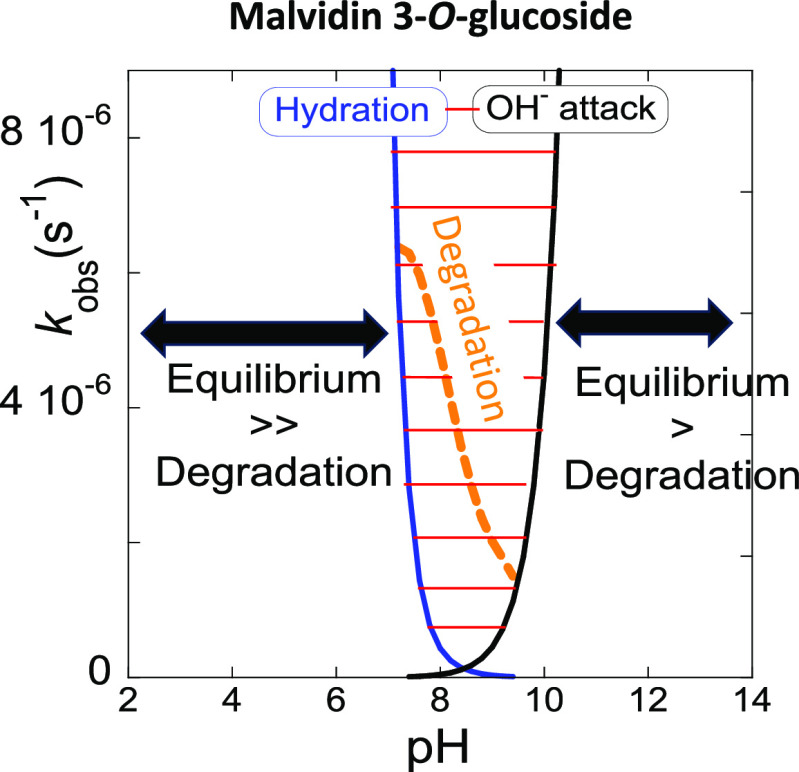

The kinetics, thermodynamics, and degradation of malvidin mono- and diglucosides were studied following a holistic approach by extending to the basic medium. In acidic conditions, the reversible kinetics of the flavylium cation toward the equilibrium is controlled by the hydration and *cis*–*trans* isomerization steps, while in the basic medium, the OH^–^ nucleophilic addition to the anionic quinoidal bases is the slowest step. There is a pH range (transition pHs), between the acidic and basic paradigms, that includes physiological pH (7.4), where degradation reactions occur faster, preventing the system from reaching the equilibrium. The transition pH of the diglucoside is narrower, and in contrast with the monoglucoside, there is no evidence for the formation of colored oligomers among the degradation products. Noteworthy, OH^–^ addition in position 4 to form B4^2–^, a kinetic product that decreases the overall equilibration rate, was observed only for the diglucoside.

## 1 Introduction

Anthocyanins (ACs) are the molecules responsible for colors in most angiosperms, giving orange-red to purple-blue hues. Besides their functions in plants, such as attracting pollinators and seed dispersers or behaving as photoprotective agents by absorbing excess visible and UV light, consumption of AC-rich foods has been associated with numerous beneficial health effects owing to the multiple biological properties of these compounds (e.g., antiproliferative, anti-inflammatory, and antimicrobial properties).^1−7^

However, ACs are intrinsically unstable, as they can react *in vivo* with other biological compounds or metals^8−10^ and decompose due to enzymatic processes,^11^ within other possible decomposition routes. Nevertheless, ACs can be detected in native or metabolized forms in urine.^12,13^

Most of the studies about ACs in the current literature have been performed at low pH values where most ACs are mainly present in their flavylium cationic equilibrium form.

In a previous work, Dangles et al.^14^ studied the degradation rates of the cyanidin-based ACs extracted from red cabbage at pH = 7 and pH = 8 and used reverse pH jumps back to the flavylium cation to control the AC degradation as a function of time. In the present work, we established that the study of the AC’s reversible and irreversible processes at higher pH values, including the physiologic pH (7.4), as well as the stability of the blue color in some flowers, where the vacuoles pH is slightly basic,^15^ requires the extension of the ACs studies to the alkaline medium (holistic approach). Malvidin-3-*O*-glucoside (M3G) and malvidin-3,5-*O*-diglucoside (M3,5diG) were selected to compare the effect of the glycosylation pattern in the thermodynamics as well as the kinetics of the reversible and irreversible chemical processes, focusing essentially on neutral and slightly basic pH values, including mechanistic clues in the degradation pathways. The extension to a basic medium is required to calculate with accuracy the rate constants for the OH^–^ nucleophilic addition.

## 2 Materials and Methods

M3G and M3,5diG were purchased from Extrasynthese ≥95%. All other chemicals were of analytical grade. pH jumps monitored by UV–vis and stopped-flow are described elsewhere.^16^

The Varian-Cary 100 Bio and 5000 spectrophotometer (Palo Alto, CA, USA) were used to record the UV–vis spectra. The stopped flow experiments were performed on an SX20 Applied Photophysics (Surrey, UK) spectrometer equipped with a PDA 1/UV photodiode array detector.

### 2.1 High-Performance Liquid Chromatography with Diode-Array Detection

The degradation of M3G at different concentrations (3.33 × 10^–5^, 2 × 10^–4^, and 1 × 10^–3^ M) was followed over time at pH 8.1 by HPLC coupled with DAD (HPLC-DAD). Stock solutions of M3G (1 × 10^–4^, 6 × 10^–4^, and 3 × 10^–3^ M) were prepared in 0.1 M HCl. Then, 1 mL of NaOH 0.1 M, 1 mL of universal buffer at pH 8.1, and 1 mL of each stock M3G solution were added to a glass vial, and the solution was analyzed over time by HPLC-DAD on a Thermo Ultimate 3000 liquid chromatograph. The decrease in the concentration of M3G was followed over time during 7 days in a 250 × 4.6 mm i.d., 2.7 μm Poroshell 120 reversed-phase C18 column (Agilent) at 25 °C. The eluents used were (A) 7.5% (v/v) formic acid in water and (B) 7.5% (v/v) formic acid in acetonitrile at a flow rate of 0.4 mL/min. The gradient consisted of 3% to 15% B in 11 min, then 25% B in 23 min, 30% B in 27 min, and then isocratic for 4 min. Detection was carried out from 200 to 800 nm in a Thermo Accela PDA detector. Due to the low concentration of M3G (3.33 × 10^–5^ M) needed to avoid self-association and the low sensitivity of HPLC analysis when compared to UV–visible spectroscopy, several aliquots (1 mL) of the degradation kinetic (pH 8.1) were taken, lyophilized, and then resuspended in 0.1 mL of 0.1 M HCl to be analyzed by HPLC to tentatively identify the degradation products.

### 2.2 LC–MS Analysis

Selected aliquots during the M3G degradation experiments were analyzed by LC–MS in order to tentatively identify the degradation products. Mass spectrometry (MS) analysis was performed using a Finnigan Surveyor series liquid chromatograph, equipped with a 250 × 4.6 mm i.d., 2.7 μm Poroshell 120 reversed-phase C18 column (Agilent) at 25 °C. The eluents used were (A) 1% (v/v) formic acid in water and (B) 1% (v/v) formic acid and 30% (v/v) acetonitrile in water at a flow rate of 0.4 mL/min. The elution gradient was performed from 20 to 100% B for 70 min; then, the column was washed with 85% B for 10 min and stabilized with the initial conditions for 10 min. The mass detection was carried out in the positive ion mode in a Finnigan LCQ DECA XP MAX (Finnigan Corp., San José, CA, USA) mass detector with an API (Atmospheric Pressure Ionization) source of ionization and an ESI (Electrospray Ionization) interface. Spectra were recorded in the positive ion mode between *m*/*z* 300 and 2000.

### 2.3 Nuclear Magnetic Resonance Spectroscopy

To evaluate the degradation process of M3G, a stock solution of commercial M3G chloride (Extrasynthese ≥95%, 6 × 10^–4^ M) was prepared in 0.1 M HCl. For sample preparation, 200 μL of 0.1 M NaOH, 195 μL of universal buffer (pH 8.1), 5 μL of internal reference TSP (2,2,3,3-*d*(4)-3-(trimethylsilyl) propionic acid sodium salt 98+ atom % D, 2 mg/mL), and 200 μL of the M3G stock solution were added to a 5 mm NMR tube. The final concentration of M3G was 2 × 10^–4^ M. The ^1^H spectra of the M3G solution were recorded over time (256 scans) at 25.00 (±0.01) °C.

Nuclear magnetic resonance spectroscopy (NMR) experiments were conducted on a Bruker Ascend 600 spectrometer, operating at a ^1^H frequency of 600.13 MHz, and equipped with a 5 mm BBO Cryogenic Prodigy Probe. All measurements were performed with standard Bruker pulse sequences at either 5.00 or 25.00 (±0.01) °C, and the spectra were processed by the Bruker Topspin software package. The concentration of M3G was set to 1.0 × 10^–3^ M and prepared in 20 mmol/L Tris-*d*_11_ buffer solution (in D_2_O, pH 8.10 ± 0.01). The ^1^H chemical shifts are given with respect to the methyl protons of tetramethylsilyl propionate, which were arbitrarily set at 0 ppm. A similar experiment was performed at 25.00 (±0.01) °C and 5° (±0.01) °C using a concentration of M3G of 1 × 10^–3^ M.

The 1D and 2D spectra of d-glucose were also recorded as a control at 5.00 or 25.00 (±0.01) °C. For that, a solution of d-glucose (1 × 10^–3^ M) was prepared in Tris-*d*_11_ buffer at pH 8.1.

The pH values of all solutions were determined on a WTW pH 320 or 508 (Weilheim, Germany) with a CRISON 5209 combined glass electrode of 3 mm diameter (Barcelona, Spain), previously calibrated with buffer solutions (pH 4, 7, and 10).

One-dimensional ^1^H spectra were acquired with a shaped pulse to suppress the water resonance using the following parameters: a spectral width of 12 ppm; a shaped pulse duration of 2.0 ms; a relaxation delay of 2.0 s; and a 90° nutation angle duration of 12.80 μs. For each individual spectrum, 128 scans were collected per FID, consisting of 32,768 complex data points. ^1^H diffusion measurements were taken using a 2D LED experiment based on bipolar gradients and an eddy current reduction delay with presaturation. A total of 64 scans of 16 data points were collected using a 90° pulse angle, a relaxation delay of 2.5 s, and a spectral width of 12 ppm. The maximum gradient strength produced in the *z* direction was 50 G/cm. The duration of the magnetic field pulse gradients (δ = 2400 μs) and diffusion time (Δ = 200 ms) were optimized in order to obtain a 5% residual signal with the maximum gradient strength. The pulse gradients were incremented from 2 to 95% of the maximum gradient strength in a linear ramp. The temperature was set and controlled to 25.00 ± 0.01 °C with a gas flow of 500 L/h in order to avoid any temperature fluctuations due to the sample heating during magnetic field pulse gradients.

### 2.4 Computational Studies

The Maestro software^17^ was used to do the conformational search of all conformers of both M3G and M3,5diG molecules. All geometries were optimized at the SMD(water)/B3LYP-D3/6-31+G(d,p)^18−21^ level of theory and using the Gaussian 09 program package.^22^ The charge distribution was analyzed by applying the NBO formalism.^23^

## 3 Results and Discussion

ACs exhibit a range of species interconnected by pH-dependent equilibria when they are in aqueous solution. To lay the groundwork for discussing the results pertaining to the basic pH region and to introduce these species, the results hitherto obtained for the acidic/neutral region are presented.

### 3.1 Acidic Medium. A Previously Described Paradigm

**Scheme 1:**
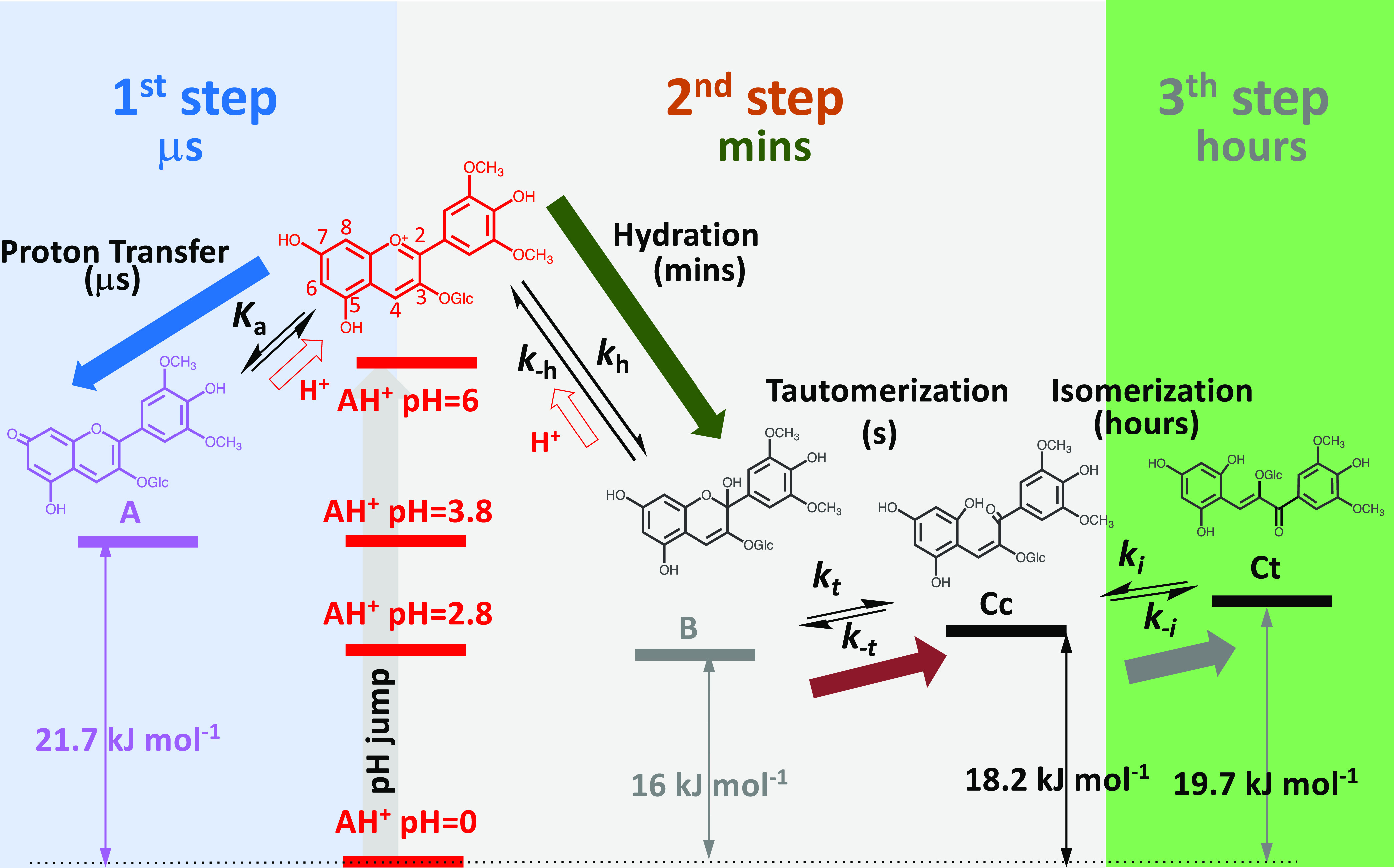
Relative Energy Level Diagram of the pH-dependent Reversible Reactions of M3G up to pH ≈ 6 This system is conveniently studied upon addition of base to equilibrated solutions of the flavylium cation (direct pH jumps). Identical schemes are followed by all ACs and related compounds.

While identified by the respective flavylium cation, ACs are not reduced to this molecule, Scheme 1.^12^ However, all species of the network converge to the flavylium cation by increasing proton concentration, which becomes the sole species, in ACs generally for pH ≤ 1, (justifying the identification of the ACs by the respective flavylium cation).^24^ Most of the ACs studies carried out on the last decades regard their behavior in the acidic medium, Scheme 1,^25^ because it is the pH domain of the vacuoles where ACs are located in most flowers (but not all) and fruits.^26,27^

The construction of the relative energy level diagram of the AC species appearing in the acidic medium, in particular the one of M3G, was previously reported in the literature,^28,29^ and it is crucial to rationalize the kinetics and thermodynamics of these compounds.

A convenient way to calculate the kinetics as well as the thermodynamics of ACs consists of adding base to equilibrated solutions of the flavylium cation (direct pH jumps) or acid to equilibrated solutions at higher pHs (or pseudoequilibrated, see below) to regenerate flavylium cations (reverse pH jumps). Except for more complex ACs,^30^ at pH ≤ 1, the flavylium cation is the sole species. When a direct pH jump, for example, to pH 6, is carried out (Scheme 1) all the other species become thermodynamically more favorable, and this constitutes the driving force to the subsequent kinetics steps. Since proton transfer is by far the fastest kinetic step (sub microseconds),^6,7^ the quinoidal base is formed and equilibrates with the flavylium cation during the mixing time of the stopped-flow (6 ms). As discovered by Brouillard and Dubois,^6^ the quinoidal base does not hydrate in acidic medium and behaves as a kinetic product that retards the hydration reaction, which occurs only via the flavylium cation to form hemiketal. In fact, the hydration rate decreases with increasing pH. The quinoidal base at pH 6 still possesses a relatively high energy level, and the system tends to evolve to the other more stable species. At the pH values reached by direct pH jumps, the hydration takes several minutes and tautomerization takes seconds. Consequently, the second step is controlled by the former, Scheme 1. The isomerization, third step, is much slower and occurs in hours, and therefore, a transient state is formed resulting from the equilibration of all species except *trans*-chalcone, the so-called pseudoequilibrium.

The equilibrium constants reported in Scheme 1 are straightforwardly calculated by means of reverse pH jumps, followed by stopped-flow and by standard spectrophotometry for *K*_i_ (see in Supporting Information Section A).^16^ The kinetics of the first step in Scheme 1 cannot be measured by stopped-flow, but the absorption spectra versus pH taken 10 ms after the jump can be collected, from which the respective acidity constants are calculated, Table 1 (see the Supporting Information, section B).

**Table 1:** Acid–Base Constants of (M3G)^43^ and (M3,5diG) Quinoidal Bases as Well as the Tautomerization Equilibrium^a^

	p*K*_a_	p*K*_A/A^–^_	p*K*_A^–^/A^2–^_
M3G	3.8	6.3	8.1
M3,5diG	3.8	7.0	

a Estimated error 10%.

Besides the equilibrium constants, direct pH jumps allow for the determination of the rate constants of Scheme 1. The mathematical expression to account for the rate constant of the second step in the acidic medium is given by eq 1.^25,31^
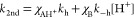
1where χ_AH_^+^ and χ_B_ are, respectively, the mole fraction of the flavylium cation in its equilibrium with quinoidal bases and hemiketal in its equilibrium with *cis*-chalcone.
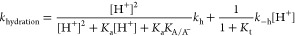
2

Fitting of *k*_hydration_, eq 2, using the data of Table 1, allows for the determination of the rate constants *k*_h_ and *k*_–h_ and by consequence the respective equilibrium constant *K*_h,_ that should be coherent with the value obtained by means of the reverse pH jumps, Table 2 (see note 1 regarding eq 2 in Supporting Information).

**Table 2:** Rate and Equilibrium Constants of Mono- and Diglucosides of Malvidin in the Acidic Medium^a^

	*k*_h_ (s^–1^)	*k*_–h_ (M^–^^1^ s^–^^1^)	p*K*_h_	*K*_t_	*K*_i_
M3G	0.08	47	2.8	0.41	0.45^16^
M3,5diG	0.33	25	1.9	0.45	0.54^44^

a Estimated error 10%.^16^

Finally, the isomerization rates and the respective equilibrium constants can be calculated from direct pH jumps, followed by standard spectrophotometry and fitting to eq 3, Table 2.
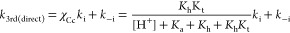
3

### 3.2 Extension to the Basic Region. The Basic Paradigm

**Scheme 2:**
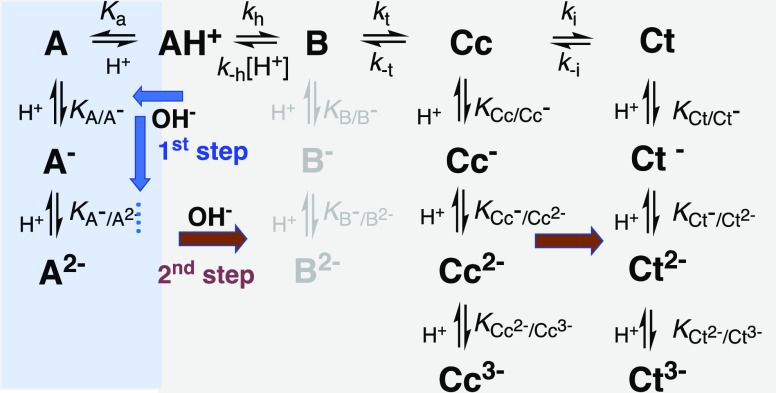
General Scheme of All Possible ACs Species That Can Be Formed as Transients or at the Equilibrium upon Extending to Basic pH Values, Together with the Definition of the Respective Equilibrium Constants In the case of the diglucoside, the species A^2–^ cannot to be formed. No spectral evidence of the hemiketal anionic forms at the equilibrium was obtained. The 2nd step is controlled by the OH^–^ nucleophilic addition since the *cis*–*trans* isomerization is much faster; see below.

The extension of direct pH jumps to the basic medium gives the possibility for the species, as shown in Scheme 1, to deprotonate in the hydroxyl substituents. In Scheme 2, all possible species that, in principle, can appear as transients or at equilibrium are presented, and the respective equilibrium constants are defined.

When a direct pH jump is extended to the basic region, the first step is still the formation of quinoidal bases, with a pH dependent mole fraction distribution defined by the acidity constants of Table 1. The second step in basic medium is due to the OH^–^ nucleophilic addition to the anionic quinoidal bases. As will be discussed below, the *cis*–*trans* isomerization is much faster than the OH^–^ nucleophilic addition, and in the basic medium, only two steps are observed, while exhibiting different kinetics in the mono and diglucosides. Except for the pH range between the acid and basic paradigms, see below, all these processes are accompanied by slower degradation kinetics.

#### 3.2.1 Malvidin-3-*O*-glucoside (M3G)

**Figure 1:**
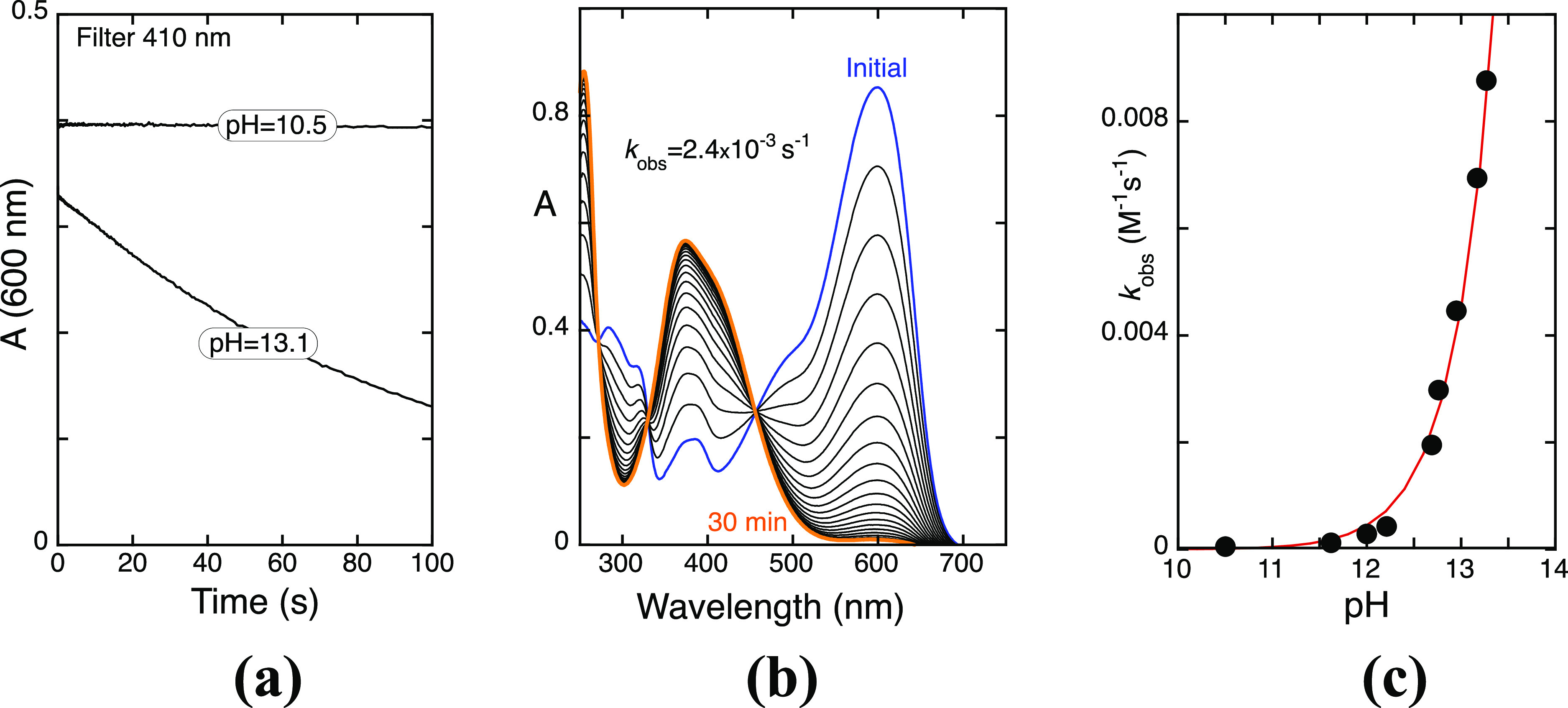
(a) Stopped-flow traces after direct pH jumps of M3G with a 410 nm cutoff filter at two representative basic pH values. See the Supporting Information, section C, for more details; (b) spectral variations of M3G (2 × 10^–5^ M) upon a direct pH jump to pH 12.8, followed by standard spectrophotometry; (c) rates of isomerization in the basic medium. Fitting was achieved for 0.045[OH^–^] s^–1^.

Direct and reverse pH jumps monitored by stopped-flow are an indispensable tool to account for the characteristic kinetic processes of the AC’s network.^25^ A series of direct pH jumps of M3G monitored by stopped-flow for 10.5 < pH < 13.5 was performed and reported in Figure 1a for two representative pH values. In this pH range, the species formed immediately after the pH jump is the dianionic quinoidal base (A^2–^), Table 1. The trace obtained at pH = 10.5 indicates that there is no reaction of A^2–^ in this time scale, while at pH = 13.1, the mono exponential decay, *k*_obs_ = 5 × 10^–3^ s^–1^, is coincident with the rate constant toward the equilibrium from A^2–^, Figure 1b,c, suggesting that the rate-determining step toward the equilibrium of M3G in basic medium corresponds to the OH^–^ nucleophilic addition, with a constant 0.045[OH^–^] s^–1^, as represented in Figure 1c.

**Figure 2:**
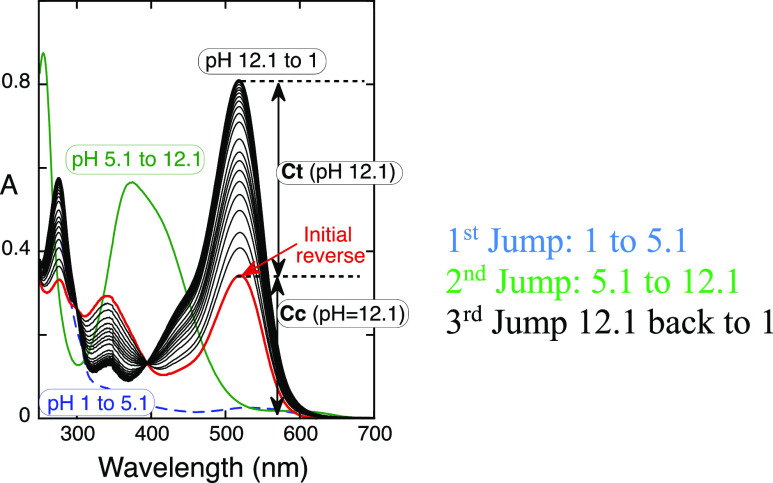
Triple sequence of pH jumps monitored by a standard spectrophotometer: blue line, final absorption spectra after a direct pH jump to pH 5.1 (pseudoequilibrium in acidic medium); green line, absorption spectra upon a direct pH jump from the solution at pH 5.1 to pH = 12.1. The absorption spectrum of the anionic *trans*-chalcone is obtained immediately after this pH jump (less than 2 min). Finally, this last solution is acidified to pH = 1. The amplitudes of *cis* and *trans* chalcones are those of the respective anionic species at pH = 12.1.

To verify this assumption, a different experiment using a triple sequence of pH jumps was designed and followed by standard spectrophotometry. A typical experiment is displayed in Figure 2. The first direct pH jump was carried to pH = 5.1, and the pseudo equilibrium was achieved in ca. 30 min (dashed blue line in Figure 2). The pseudoequilibrium of M3G consists of 6% of A, 65% of B, and 29% of Cc.^16^ A second pH jump from the pseudoequilibrium to pH 12.1 was performed, and after the collection of the first spectrum at this last pH value (green line in Figure 2), the anionic *trans*-chalcone (in equilibrium with the respective *cis*-chalcone) was already formed in less than 2 min, the time required to carry out this step. This allows for concluding that *cis*–*trans* isomerization is very fast under basic conditions. When the direct pH jump is performed directly from pH 1 to pH 12.1, the rate for the chalcone formation is 7.1 × 10^–4^ s^–1^ corresponding to a lifetime of 23.4 min. Clearly, the rate-determining step of the kinetics toward equilibrium is not the *cis*–*trans* isomerization but the OH^–^ nucleophilic addition. The last pH jump in Figure 2 consists of a reverse pH jump from pH 12.1 to pH = 1. The amplitude of the initial spectrum obtained at pH 1 corresponds to the amount of anionic *cis*-chalcone formed at pH 12.1 (no evidence for anionic hemiketal was obtained at pH 12.1 by stopped-flow) that turns into the flavylium cation in a few seconds. The amplitude of the flavylium recovery kinetics (at pH = 1) is due to the fraction of anionic *tran*s-chalcone at pH = 12.1, which needs to undergo slower isomerization to *cis*-chalcone before flavylium formation.

#### 3.2.2 Malvidin-3,5-*O*-diglucoside (M3,5diG)

**Figure 3:**
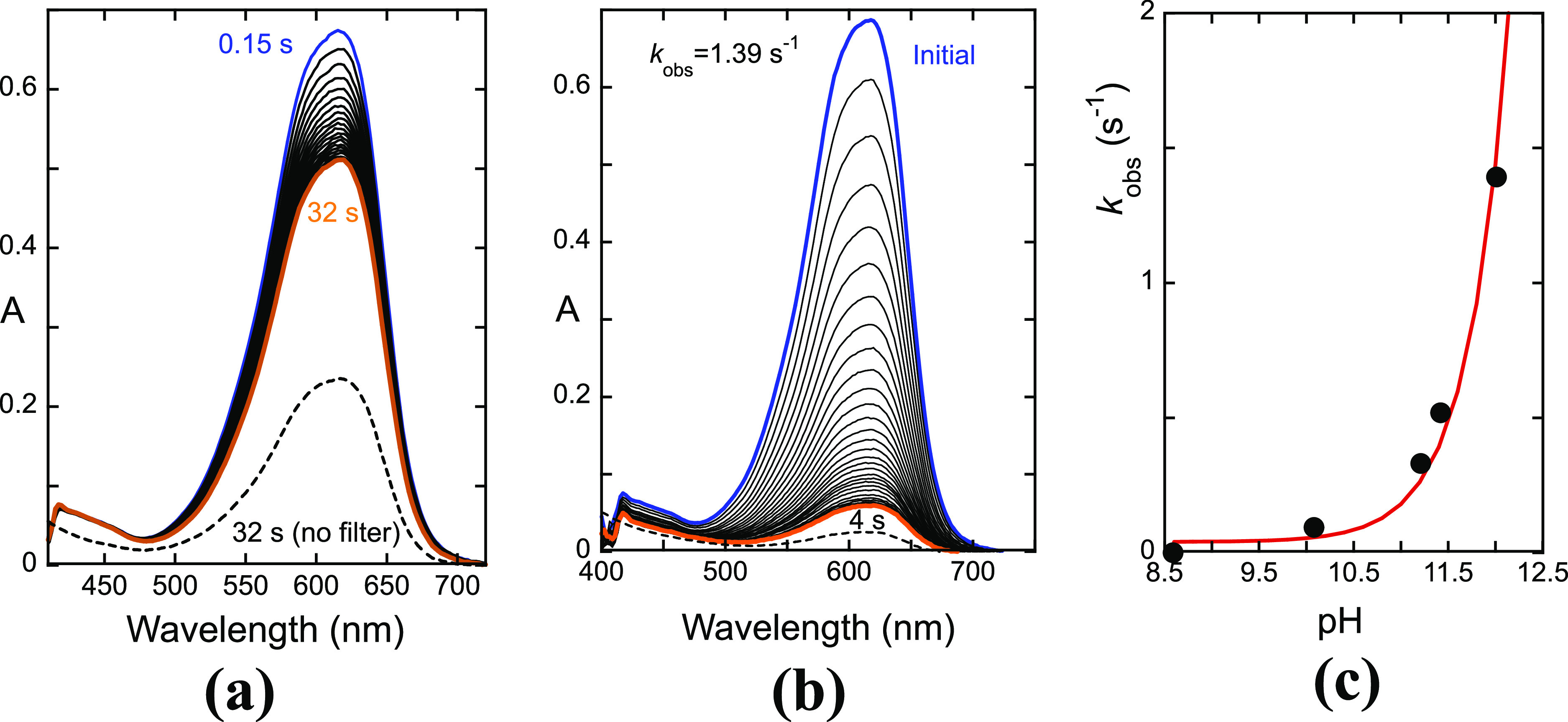
(a) Spectral variations of M3,5diG (1.6 × 10^–5^ M) after a direct pH jump to pH 10.1 monitored by stopped-flow using a 410 nm cutoff filter; (b) the same as (a) for pH 12; (c) rate constants to reach the transient equilibrium in M3,5diG measured by stopped-flow (faster step). These rate constants are directly proportional to the hydroxyl concentration according to the formula 140[OH^–^] s^–1^.

There is a large difference between M3G and M3,5diG regarding the quinoidal base formed immediately after a direct pH jump in the basic medium. In the last compound, only the mono anionic quinoidal base (A^–^) can be obtained, Table 1. The spectral variations of M3,5diG monitored by stopped-flow after direct pH jumps are shown in Figure 3a,b, for two representative pH values. Inspection of these figures reveals the existence of a transient pre-equilibrium between the anionic quinoidal base and another species (that below we identify as B4^2–^, resulting from the OH^–^ attack in position 4 of the quinoidal base) achieved in 32 and 4 s, respectively, at pH 10.1 and pH = 12. Moreover, the fraction of the anionic quinoidal base decreases by an increase in pH. Representation of the rate constants to reach this pre-equilibrium can be fitted with 140[OH^–^] s^–1^, Figure 3c. Such an equilibrium is not observed in M3G.

**Figure 4:**
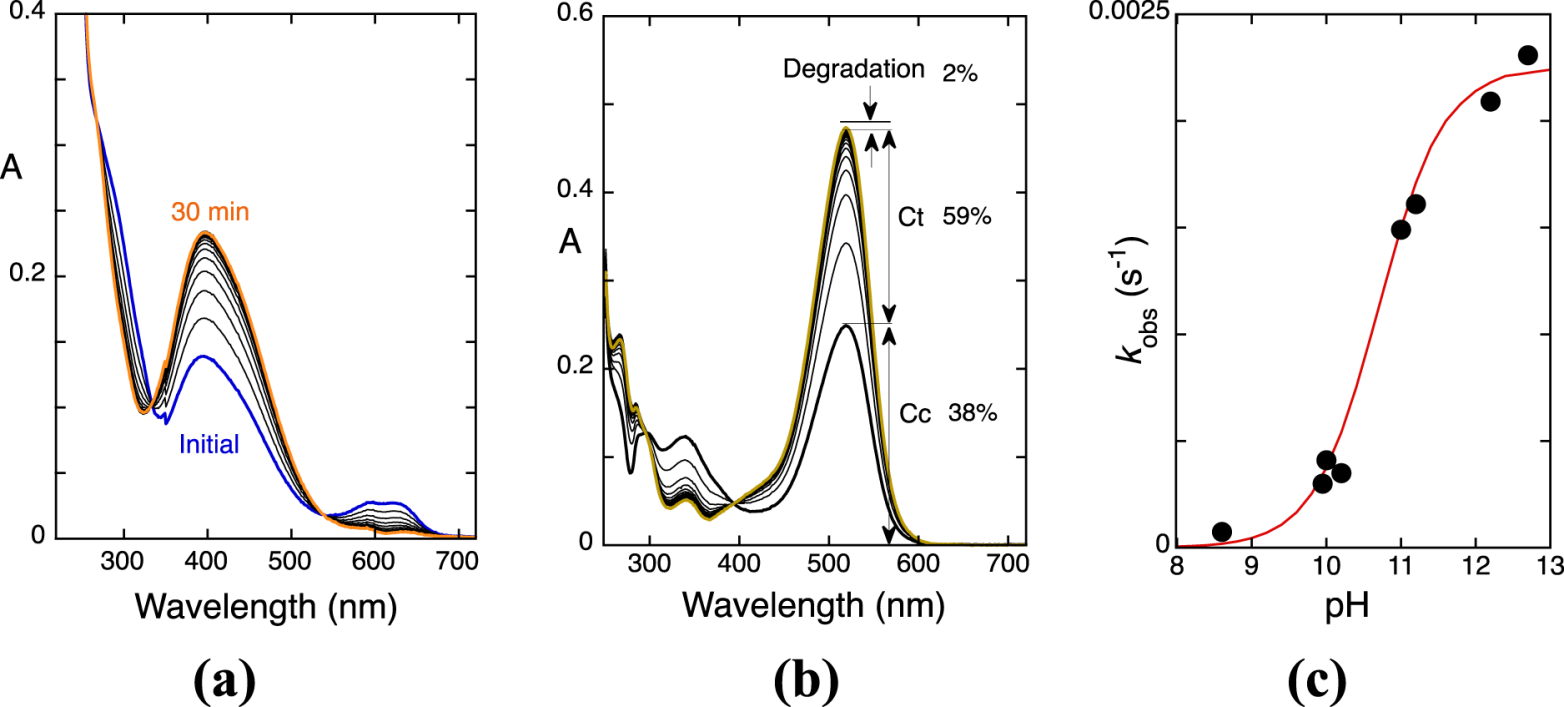
(a) Spectral variations of M3,5diG (1.6 × 10^–5^ M) upon a direct pH jump to pH 12.0; (b) reverse pH jump from the solution at pH 12 after 30 min (shown in (a) to pH 1; (c) rate constants of the isomerization step versus pH. Fitting was achieved for an inflection point at pH = 10.7.

The direct pH jumps monitored by a standard spectrophotometer, Figure 4, complement the above-described stopped flow experiments. A direct pH jump to pH 12.0 is represented in Figure 4a. The initial absorption reflects the pre-equilibrium and is reported in Figure 3 (red trace in Figure 3b, achieved after 4s). Please note that the mole absorption coefficient of the quinoidal base (42,000 M^–1^ cm^–1^ at 618 nm) is much higher than that of the anionic chalcones (average value 15,000 M^–1^ cm^–1^ at 378 nm according to Figure 4a). However, in Figure 4a, a small decrease in the absorption of the anionic quinoidal base leads to a large increase in absorption of the anionic chalcones, despite the smaller mole absorption coefficient of the latter one. This behavior can be explained by considering the colorless B4^2–^ species in equilibrium with the anionic quinoidal base (see below Section 2.2) that behaves as a kinetic product evolving through the anionic quinoidal base, to the thermodynamically stable chalcones (eqs 4 and 5). Kinetically, this product behaves like the neutral quinoidal base in the acidic medium (i.e., as a kinetic reservoir). A reverse pH jump in the solution at pH 12 after 31 min back to pH 1 permits calculation of the fraction of anionic *cis* and *trans* chalcones as well as the fraction of degradation by comparison with the expected absorbance of the flavylium cation. This experiment shows that, in this time frame, the system reaches the equilibrium without being significantly affected by the degradation processes. The pH-dependent observed rates toward equilibrium of the M3,5diG are represented in Figure 4c.

The triple sequence of pH jump experiments reported in Figure 2 for M3G was repeated for M3,5diG, and the results were identical. The *cis*–*trans* isomerization in the basic medium is a very fast process in both compounds, implying the rate-limiting addition of OH^–^ to the anionic quinoidal base. The explanation for the sigmoidal-shaped *k*_obs_ vs pH shown in Figure 4c for the appearance of the anionic *trans*-chalcone, in the case of M3,5diG, is thus the existence of an equilibrium defined by eq 4 that decreases the mole fraction of the anionic quinoidal base and retards the OH^–^ addition kinetics toward the equilibrium.
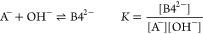
4

A mass balance allows for the determination of the mole fraction of the anionic quinoidal base, χ_A^–^_, in its equilibrium with the species B4^2–^, and the rate toward the equilibrium is given by eq 5.
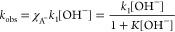
5

The fitting reported in Figure 4c was achieved for *K* = 2.04 × 10^3^ M^–1^ and *k*_1_ = 4.5 M^–1^ s^–1^, showing that the addition of OH^–^ to the anionic quinoidal base in M3,5diG is much faster than to the dianionic quinoidal base of the M3G, in agreement with a lower electrophilic character of the latter. Considering that the anionic quinoidal base behaves as a Lewis acid, a p*K*_a_ = −log(*K* × *K*_w_) = 10.7 can be calculated, which corresponds to the inflection point of Figure 4c.

Summarizing, in both compounds, the OH^–^ nucleophilic addition is the rate-limiting step toward the equilibrium in the basic medium, but in M3,5diG there is the additional restriction imposed by the pre-equilibrium between A^–^ and B4^2–^ that decreases the fraction of the anionic quinoidal base available to react with OH^–^, see eq 5.

**Scheme 3:**
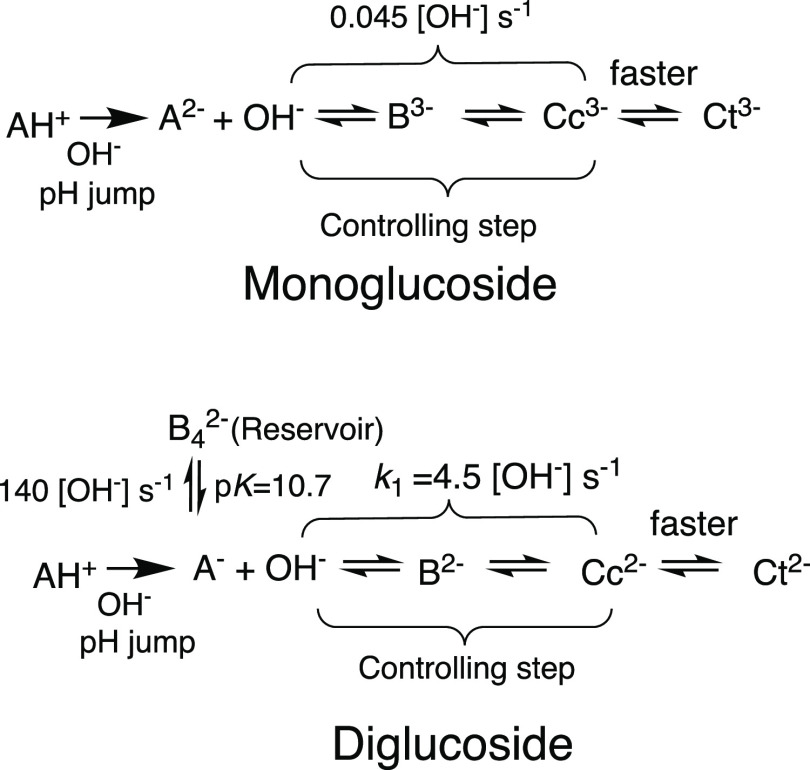
Proposed Sequence of Reactions for the Kinetics toward the Equilibrium in Both Malvidin Mono and Diglucoside in the Basic Medium The species B4^2–^ was identified below in Section 3.3 (kinetic signatures) and 2.2.1 (^1^H NMR).

The sequence of the chemical reactions in the basic medium is summarized in Scheme 3 for both compounds.

### 3.3 Mono Versus Diglucoside. The Kinetic Signatures

As previously demonstrated,^16^ reverse pH jumps monitored by stopped-flow provide a powerful tool to investigate the ACs and related compounds systems. They are based on the fact that for pH ≤ 1, the hydration becomes faster than tautomerization (change of regime).^32^ The main advantage of this approach relies on the fact that the different species of the ACs network are converted into the flavylium cation at different rates. In other words, each species displays a specific kinetic signature that allows for its accurate identification as well as quantification. For M3,5diG, for example, after a reverse pH jump from the pseudoequilibrium to [HCl] 0.5 M (sufficiently acidic to have a good separation between the traces of hydration and tautomerization), the kinetic signature of the hemiketal (B2) is 8.7 s^–1^, while that of *cis*-chalcone is 2.3 s^–1^ and that of Ct is 1.8 × 10^–4^ s^–1^ (in this last case requiring a standard spectrophotometer), see Supporting Information, section A.

**Figure 5:**
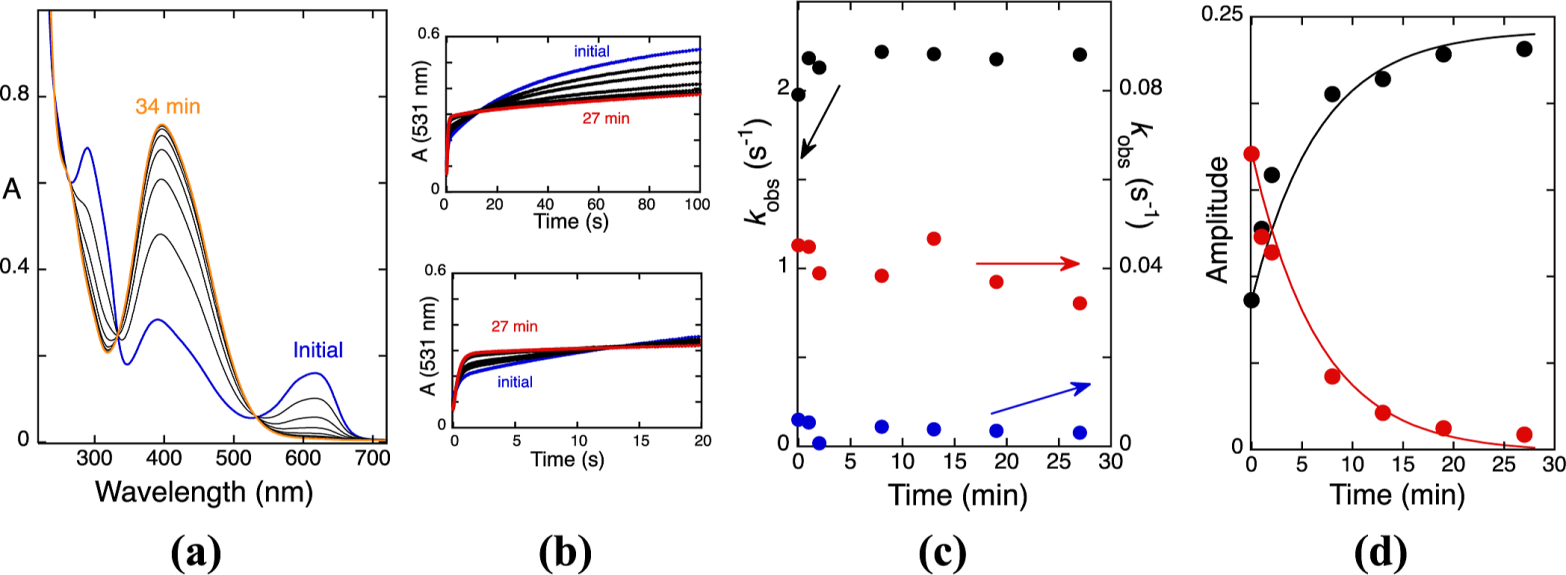
(a) Spectral variations of M3,5diG after a direct pH jump to pH = 11.3. Fitting was achieved with a monoexponential with rate constant 2.5 × 10^–3^ s^–1^ at pH = 11.5; (b) Reverse pH jump to [HCl] = 0.5 M with a cutoff filter of 435 for the following times of (a) 0, 1, 2, 8, 13, 19, and 27 min; fitting was achieved for each of these times with a triexponential; (c) representation of the rate constants; (d) representation of the amplitudes of (b).

The existence of the kinetic product B4^2–^ for M3,5diG, proposed in Scheme 3 was further supported by carrying out a sequence of reverse pH jumps, as reported in Figure 5. A direct pH jump to pH 11.3 followed by a standard spectrophotometer was performed: 3 mL of this solution was immediately transferred to the spectrophotometer cell and another 3 mL to the stopped-flow syringe (this volume was enough to carry out several stopped-flow shots to [HCl] = 0.5 M). For the same intervals of time 0, 1, 2, 8, 13, 19, and 27 min, the standard spectrophotometer spectrum, Figure 5a, and the stopped-flow shot back to [HCl] = 0.5 M were performed simultaneously. Representation of the stopped-flow traces at 531 nm (flavylium cation absorption) is shown in Figure 5b. Three kinetic processes were observed. The faster process exhibits the kinetic signature of *cis*-chalcone (no trace of the hemiketal B was present). The next kinetics has a rate constant of 0.04 s^–1^, and both are clearly identified in the time scale of 20 s, Figure 2b bottom. A third and slower kinetics, Figure 5b up, has the kinetic signature of the *cis*–*trans* isomerization. In Figure 5c, the rate constants of the three traces are represented for all intervals of time. The trace with rate constant 0.04 s^–1^ was identified for the first time in this experiment and is attributed to the hemiketal B4. Representations of the amplitudes of B4^2–^ and the anionic *cis*-chalcone versus time (Figure 5d) were obtained from the triexponential fitting (Figure 5b), where the lowest rate constant, 0.0018 s^–1^, is coincident with the isomerization rate of this compound.

**Scheme 4:**
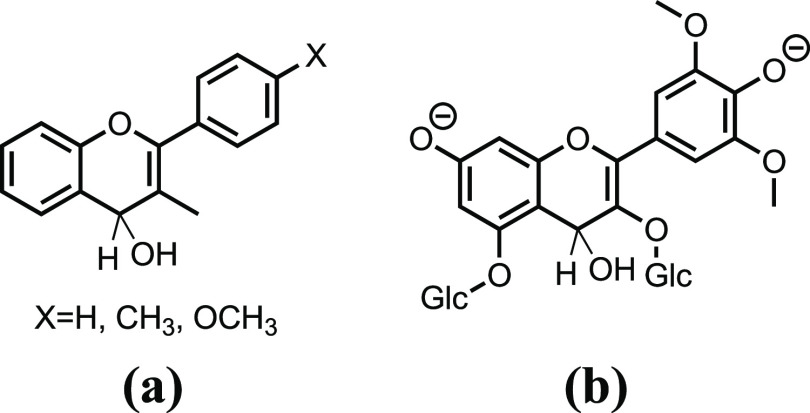
(a) Chemical Formula of the B4 Adduct Reported by McClelland and Gedge;^33^ (b) Proposed Chemical Formula for B4^2–^ Species of M3,5diG

The B4 adduct was previously reported in the 1980s by McClelland and Gedge upon a reverse pH jump from equilibrated solutions at moderately acidic pHs to pH 1, Scheme 4a. B4 results from the flavylium cation hydration in position 4 and is formed together with hydration in position 2 (to give hemiketal, B). However, in contrast to B, B4 is a kinetic product because it does not give directly *cis*-chalcone.^33^ The system evolves toward equilibrium only from B2. B4 was exclusively observed in synthetic flavylium compounds lacking hydroxyl substituents. In the case of ACs and other flavylium cations bearing hydroxyl substituents, B4 is not detected in the acidic medium because quinoidal base formation is by far much faster than any hydration reaction in position 2 or 4.

#### 3.3.1 Experimental Evidence of B4^2–^ by NMR

As shown above in Figure 3, the formation of B4^2–^ in equilibrium with A^–^ occurs in a few seconds, preventing its raising kinetics from being followed by a technique other than stopped flow. However, it was possible to monitor the kinetics of this species disappearance using NMR (600 MHz) as well as a standard spectrophotometer, carrying out both experiments with the same concentration [M,3,5diG] = 2 × 10^–4^ M and lowering the temperature to 5 °C to decrease the rate of B4^2–^ disappearance. A direct pH jump to 11.9 in the above-described conditions, monitored using a standard spectrophotometer, indicates that ca 80% of M3,5diG was converted in B4^2–^, Figure S5 in Supporting Information section D)

**Figure 6:**
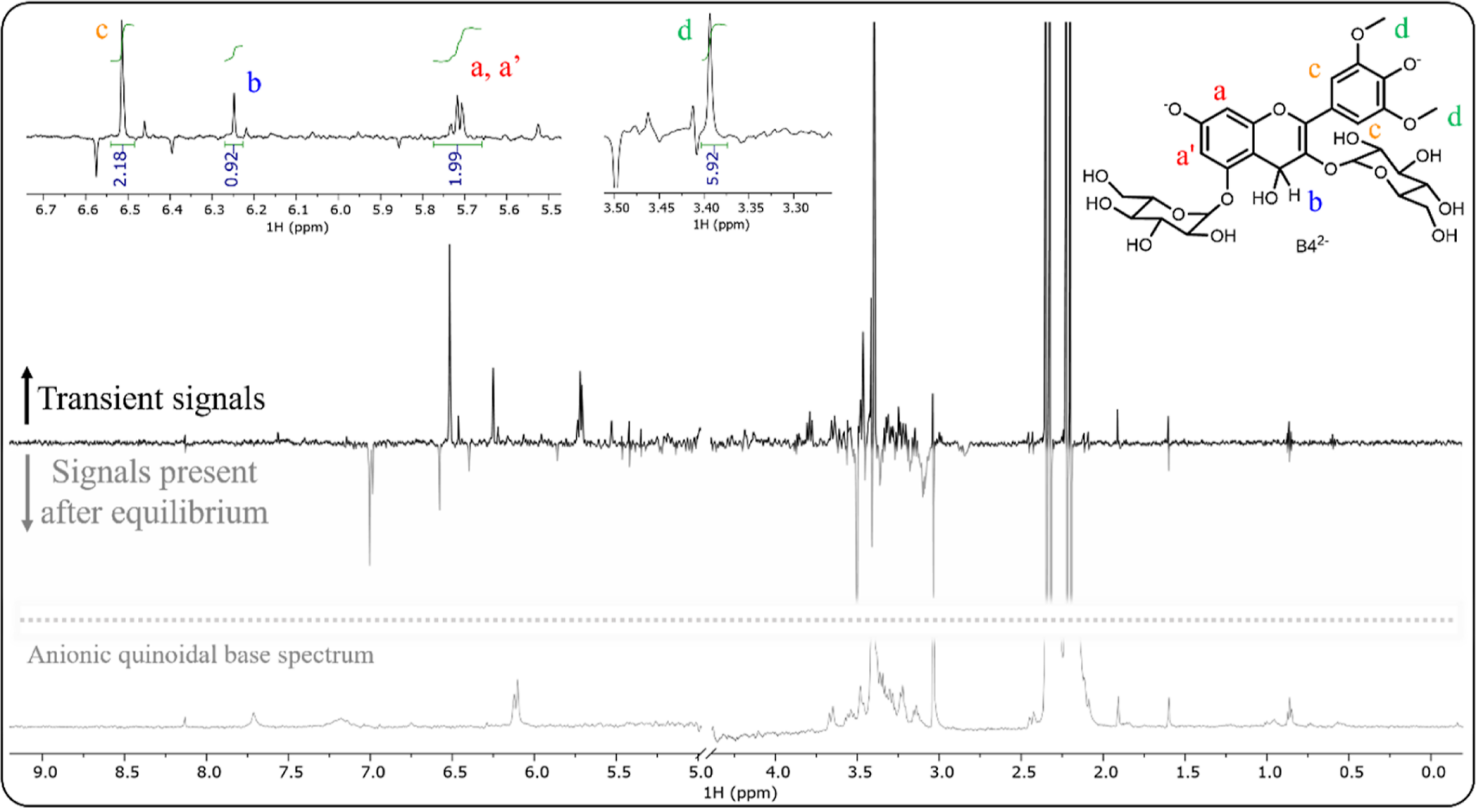
Difference in the ^1^H NMR spectrum of M3,5diG (2.0 × 10^–4^ M) between the spectra ca. 15 min and ca. 24 h after a direct pH jump from pH = 1 to pH = 11.9. The positive signals are the ones present in the beginning and absent in the end and vice versa for the negative ones. The bottom part of the image shows the ^1^H NMR spectrum of the anionic quinoidal base for comparison. The small positive peaks are those of the minor anionic quinoidal base at ca. 15 min.

Knowing this behavior, the above experiment was replicated by monitoring the evolution of the system by ^1^H NMR spectroscopy, using 10% D_2_O to allow for the locking of the magnetic field but otherwise in the same conditions, including the same concentrations of AC and buffer. With suppression of the water signal (using the NOESY 1D pulse sequence), the sequence of clear spectra over the course of ca. 24 h was obtained, Figure S6 in Supporting Information section D. In this experiment, it is clear that there is a set of signals present at its highest intensity in the beginning and immediately starts to decrease in intensity. Also, there is a set of signals that can be attributed to the chalcones since they are increasing with time and finally another set that remains constant throughout the experiment, some of which we know that belong to the buffers used. The last spectrum (after ca. 24 h) was subtracted from the first one and the resulting difference spectrum, Figure 6, clearly shows the set of signals belonging to the transient species in agreement with our hypothesis of it being B4^2–^ due to their multiplicity and integration.

To discard the fact that these signals could belong to the anionic quinoidal base, a second pH jump monitored by ^1^H NMR was made, this time from pH 1 to 8.7, replicating the experiment previously followed with UV–vis spectroscopy. A sequence of spectra following the pH jump was acquired, and as expected from the absorption data, no significant changes were observed. In Figure 6, the first spectrum is shown for comparison, and it is clear that it does not correspond to the set of transient signals, thus reinforcing our assumption that they belong to B4^2–^.

#### 3.3.2 Computational Studies

**Figure 7:**
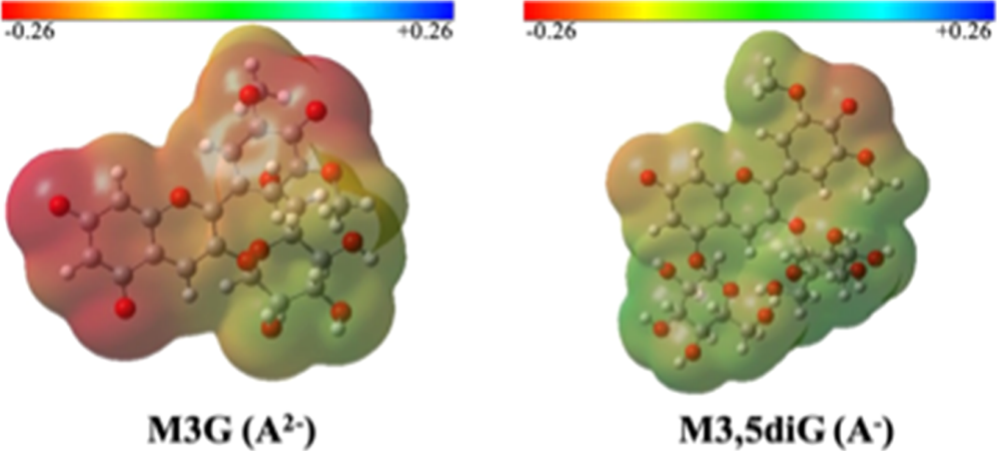
ESP map of the thermodynamically most favored geometries of M3G (A^2–^) and M3,5diG (A^–^) obtained at the SMD (water)/B3LYP-D3/6-31+G(d,p) level of theory.

The question is why in the basic medium the OH^–^ attack occurs in A^–^ of M3,5diG but not in A^2–^ of M3G. To rationalize the above experimental observations, the molecular structures of the dianionic quinoidal base of M3G and the monoanionic M3,5diG have been analyzed using quantum mechanics calculations. Table S2, in Supporting Information section E, summarizes the charge distribution of some key atoms as well as the HOMO/LUMO energy data for the most thermodynamically stable conformers of each molecule. Analysis of the charge distributions in the AC moiety indicates that the presence of the second glucose unit on the O5 atom in M3,5diG increases (makes less negative) the charge of the O5 and C6 atoms by 0.274 and 0.127 au, respectively. Indeed, a significant difference is observed in the overall charge of the AC moiety in the diglucoside, which amounts to +0.476 au. This distinct charge distribution is remarkedly notorious in the comparison of the electrostatic potential energy (ESP) maps for both molecules, Figure 7. Considering the proximity between the O5 and C4, the excess of negative charge in M3G disfavors the nucleophilic attack of HO^–^ on C4 and subsequent formation of the B4^2–^. Noteworthy, no significant differences are observed in the HOMO/LUMO energies of the two compounds, which suggests that they do not significantly interfere with the different reactivities toward nucleophiles at the C4 position.

### 3.4 Degradation Rates

The irreversible process that conduces to the ACs degradation in acidic or basic solutions can be studied by a sequence of pH jumps as follows: (i) direct pH jump from equilibrated solutions of flavylium cation to the desired pH, (ii) stand the solution for a given time (delay time); (iii) carry out a reverse pH jump to pH ≤ 1 and wait the necessary time to the flavylium cation reach the equilibrium. The degradation for each delay time is measured by means of eq 6, where *A*_initial_ is the initial absorbance of the flavylium cation, prior to step (i) and *A*_after delay_ is the absorbance after step (iii).
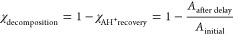
6

**Figure 8:**
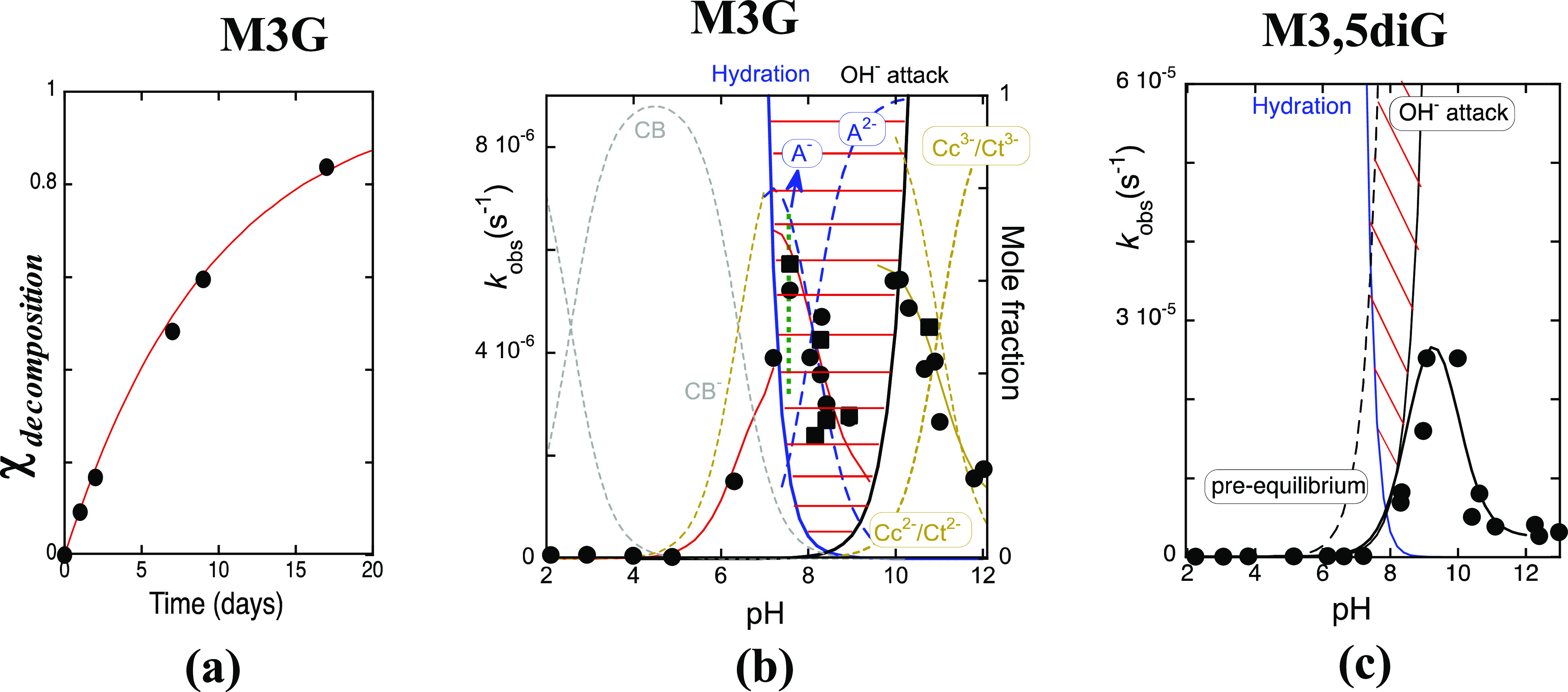
(a) Mole fraction of the degradation processes upon a pH jump of M3G (2 × 10^–5^ M) at pH = 6.3 calculated at 520 nm by means of eq 6 versus delay time. The kinetics is monoexponential with rate constant *k*_degradation_ = 1.5 × 10^–6^ s^–1^; (b) (λ) rate constants of the M3G colored species disappearance (includes the degradation products exhibiting an absorption spectra similar to M3G) versus pH in acidic and basic media, calculated as in Figure 8a, p*K* of the equilibrium between dianionic chalcones and dianionic chalcones ≈ 11; (ν) rate constant for the kinetics of quinoidal base disappearance. This figure completes and corrects the previous one reported in ref (24); (c) (λ) degradation rate constants of M3,5diG (1.3 × 10^–5^ M), calculated as in Figure 8a.

Representation of χ_decomposition_ for different delay times allows for the determination of the degradation rate, as exemplified in Figure 8a for M3G. The rate constants of the measured degradation processes versus pH are represented in Figures 8b,c, respectively for M3G and M3,5diG, black circles (●). When colored dimers and oligomers are formed in significant amounts, as in the case of M3G, the degradation rate is lower than the one measured by HPLC (see below and Supporting Information section F).

### 3.5 Transition pHs (that Include the Physiological pH)

In a previous work,^24^ it was reported that for monoglucosilated ACs, the transition pHs (include the physiological pH, in the context of this work pH = 7.4) are delimited by the hydration reaction and the OH^–^ nucleophilic addition, second step, respectively in acidic and basic media, Figure 8b. In this pH range, the equilibrium is not achieved; only anionic quinoidal bases are present (together with the respective irreversible products, some of them absorbing in the visible, see below) and no *trans*-chalcone is detected, upon reverse pH jumps back to pH ≤ 1.^24^ The fact that in the transition pHs of M3G, the degradation rates follow the mole fraction distribution of species A^–^, Figure 8b, suggests that the degradation pathways are essentially initiated from this species. In basic medium (out of the transition pHs), the degradation rate follows the mole fraction distribution of the anionic chalcones (the only observed species), suggesting a OH^–^ attack to these species, which is less efficient for the more negatively charged anionic chalcones due to their higher electrostatic repulsion.

M3,5diG behaves differently, as shown in Figure 8c. The transition pHs, also delimited by the hydration reaction and the OH^–^ nucleophilic addition, are narrower in comparison with M3G. The transient equilibrium that forms B4^2–^ (traced line in Figure 8c) is reached in almost all the pH intervals, and there is not a net separation between the kinetics toward the equilibrium and the degradation rate. On the other hand, above the transition pHs, the kinetics toward the equilibrium is always faster than the degradation, Figure 8c, following the same trend of M3G.

It should be also emphasized that in contrast with acidic and transition pHs, the degradation kinetics of the diglucoside in the basic medium is faster than that of the monoglucoside, Figure 8c (inversion of relative reactivity). This behavior was confirmed by following the kinetics by HPLC at pH = 9. After 50 h, the M3G peak is reduced 65%, while M3,5diG almost disappeared, Figure S7b in section F of Supporting Information.

A probable explanation for this behavior is the faster formation of the anionic chalcones (for pH < 12.5) of the diglucoside, see Figure S7c in section F of Supporting Information, as well the formation of B_4_^2–^. The kinetics at the physiological pH for the mono- and diglucoside are presented in more detail in Supporting Information, section F. As in the acidic medium, the disappearance of the monoglucoside at physiological pH is faster than the one of the diglucoside.

### 3.6 Identification of Degradation Products at the Transition pHs

It is worth mentioning that there is a severe limitation when comparing the kinetic constants calculated by analytical techniques that require relatively higher concentrations, with spectrophotometry, due to the well-known self-aggregation of ACs.^34^ This is particularly significant for the analysis of the degradation products. Herein, we limited our investigation to the transition pH values signed by the red traces in Figure 8.

**Figure 9:**
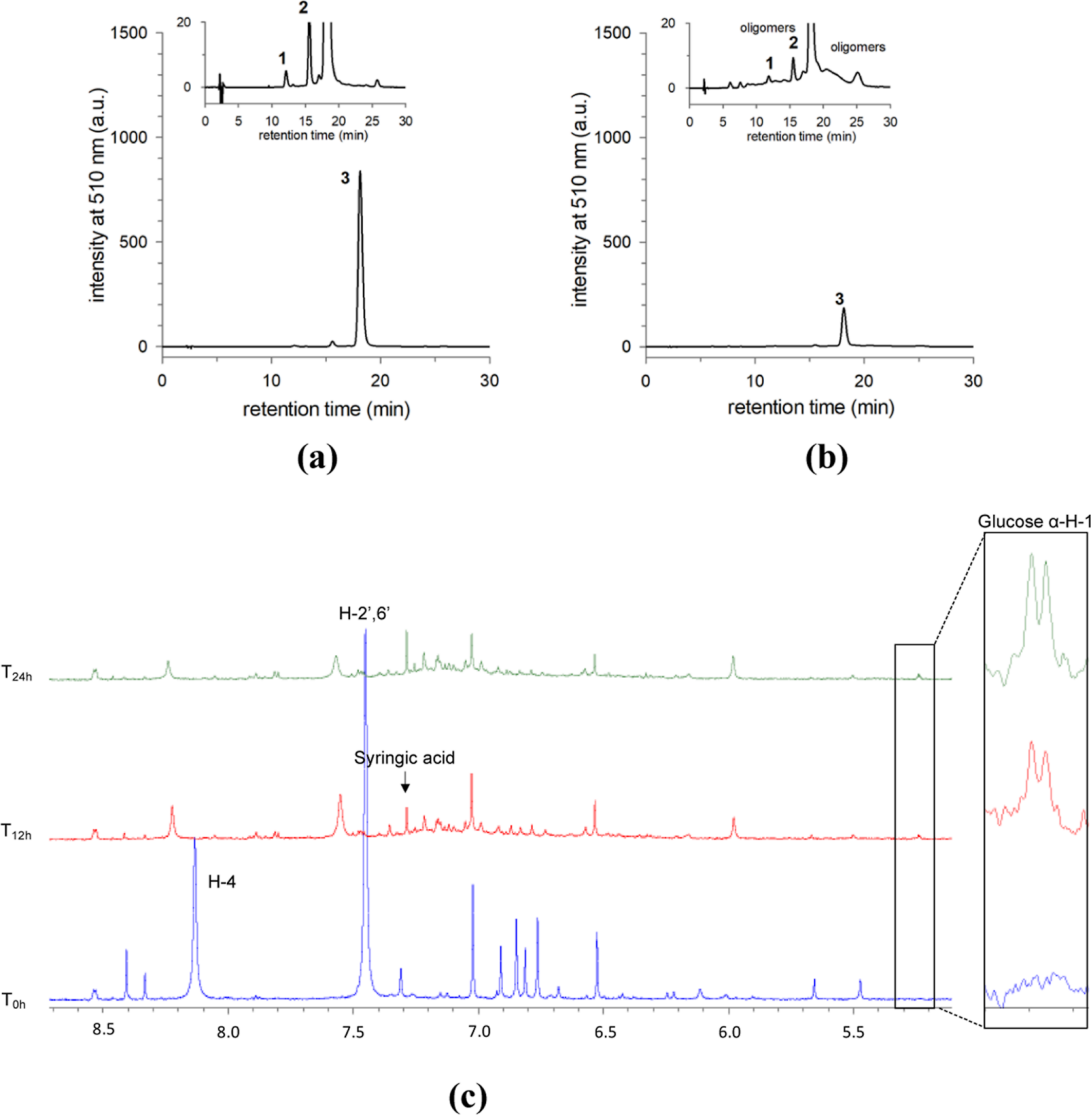
HPLC chromatograms for representative transition pHs of: (a) M3G solution (1 × 10^–3^ M, pH 8.1) at the end of 2 h; (b) and 24 h. Peak 1 corresponds to malvidin-3-*O*-glucoside dimer, peak 2 corresponds to petunidin-3-*O*-glucoside already present in M3G standard), and peak 3 corresponds to malvidin-3-*O*-glucoside; (c) ^1^H NMR spectra in the aromatic region of M3G at 1 × 10^–3^ M, pH 8.1 versus time. Spectra were acquired at 25 °C.

Several aliquots of the aqueous solution of M3G, 1 × 10^–3^ M at pH = 8.1 were taken over time and analyzed by HPLC-DAD and HPLC-MS, Figure 9a,b. Since the pH observed during the elution of the HPLC is very acidic (pH < 1), quinoidal bases and *cis*-chalcone (independently on their protonated state) revert to the respective flavylium cation, while *trans* chalcones and all irreversible products appear in their protonated forms.

It can be observed from the HPLC of Figure 9 that apart from the M3G flavylium cation (peak 3) and petunidin 3-*O*-glucoside (already present in the M3G standard, peak 2), new chromatic peaks in the visible region of the spectrum start to appear after 2 h (Figure 9a). By increasing the incubation time, the number of peaks increases significantly, creating a hump, as shown in Figure 9b, after 24 h. The formation of colored products at the physiological pH was corroborated during the disappearance of A^–^, see Supporting Information Section F, Figure S7a.

^1^H NMR studies have been reported to elucidate the different species resulting from the reversible equilibrium reactions of ACs.^30,35−37^ However, they can be extended to the basic medium not only for the characterization of the reversible species but also for the degradation products. Figure 9c shows that right after the dissolution of M3G, 1 × 10^–3^ M, at pH = 8.1, besides the signals assigned to the anionic quinoidal bases, several unidentified aromatic resonances appeared in the spectrum, which could not be attributed to either syringic acid or any other conventional degradation products.^25,38^ From the latter, only syringic acid was detected at the end of 12 h of incubation, as the same as the anomeric ^1^H signal of free α-d-glucose. These assignments were supported by DOSY and chemical shift referencing (Table S3, Figure S9, and Figure S10, Supporting Information section G). It thus becomes clear that though at pH 8.1, the anionic quinoidal base does become deglycosylated and degraded into syringic acid, these pathways cannot entirely account for the rapid kinetic decay observed by both NMR and HPLC-DAD.

**Scheme 5:**
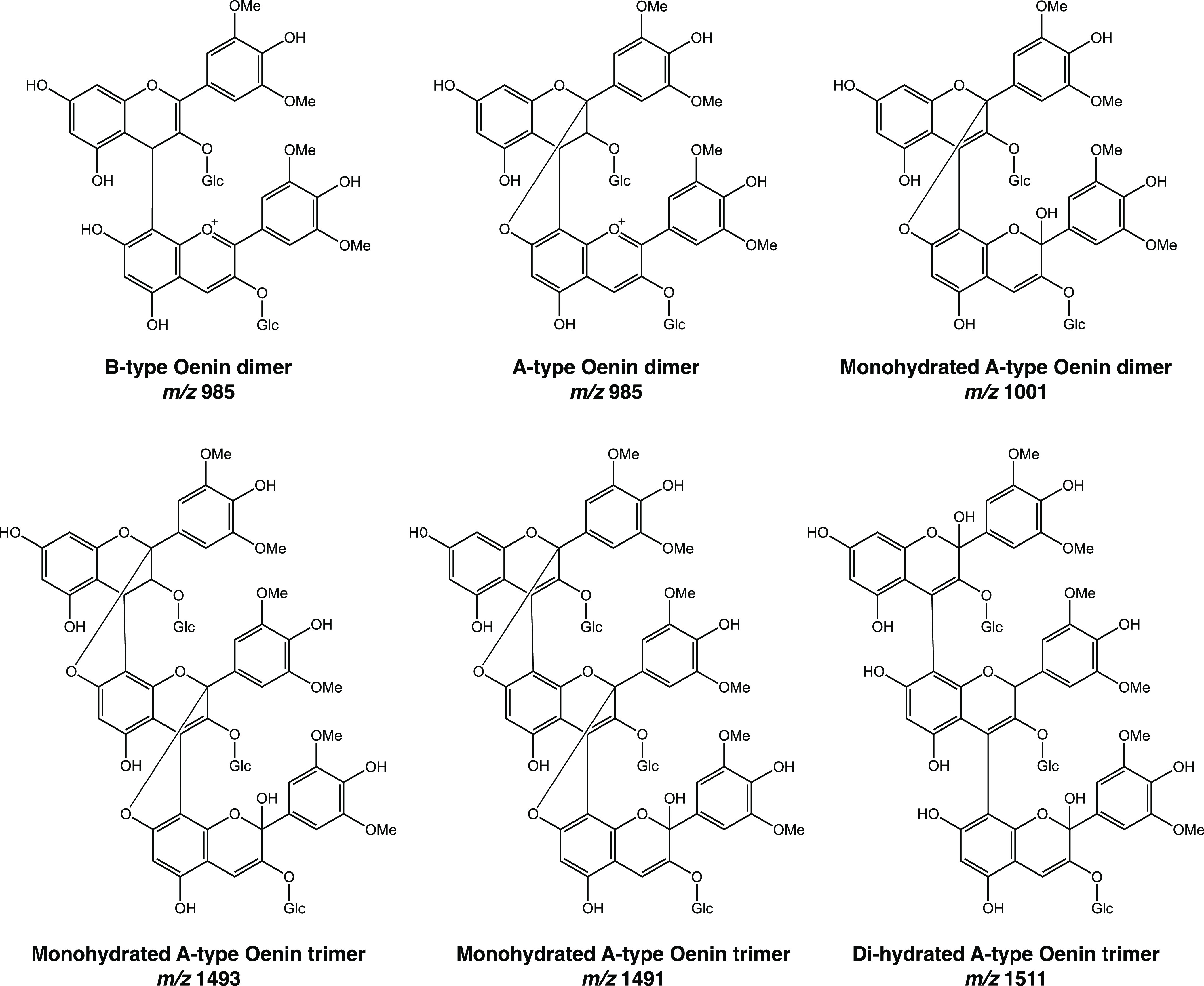
Putative Structures for Degradation Products of M3G Based on LC–MS/MS Data

The analysis by LC–MS in the positive ion mode showed the presence of different ion masses, Table 3, comprising similar fragmentation patterns such as −162, and −18 amu corresponding to the loss of glucose moieties and water. Ion masses at *m*/*z* 985 and 1001 were observed in the first stages of degradation. After 1 day at pH 8.1, ion masses at *m*/*z* 985, 1,001, 1,017, and 1493 were detected. Based on the UV–visible spectrum, ion mass, and respective fragmentation pattern, the new peaks were assigned to oligomers formed during the compound degradation. This is not the first time that AC dimers and oligomers have been described to occur in nature. Vidal et al.,^39^ Salas et al.,^40^ and Alcalde-Eon et al.,^41^ reported these AC-derivatives in grapes and wines based on the MS data. Later, Oliveira et al.,^42^ using NMR confirmed the presence of an A-type trimer in a young red wine. The putative structures of the detected ion masses during M3G degradation are listed in Scheme 5.

**Table 3:** Ion Masses in the Positive Ion Mode of the Degradation Products Formed During Degradation under Slightly Basic Conditions

compound	[M^+^]	[MS^2^]	[MS^3^]	possible identity
1	199			syringic acid
2	985	823	661	dimer 1
3	985	823	661	dimer 2
4	1001	983; 839; 677		monohydrated dimer
5	1003	985; 823		monohydrated dimer
6	1017	855; 693		
7	1511	1349; 1493; 1331		dihydrated trimer
8	1491	1329; 1167; 1005		monohydrated trimer
9	1493	1331; 1169		monohydrated trimer

Additional ion masses corresponding to hydrated and dihydrated forms of dimers and trimers were also detected as degradation products.

At low concentrations of M3G (3.3 × 10^–5^ M), oligomerization still occurs, although to a lesser extent when compared with the more concentrated solution (1 × 10^–3^ M).

Typical AC degradation mechanisms involve the loss of glucose from M3G yielding malvidin aglycone and then C3–C4 cleavage to give syringic acid (ring B) and 2,4,6-trihydroxybenzaldehyde (2,4,6-THB) which in oxidative conditions can yield the respective acid.^38^ The presence of 2,4,6-THB and syringic acid was observed during the degradation of M3G at slightly basic conditions. However, the concentration of syringic acid after the total degradation of M3G (∼7 days) corresponds to only about 10% of the initial concentration of M3G, which once more indicates that this pathway is not the main one involved in this irreversible process.

The formation and degradation of the dimer were further supported by kinetic experiments conducted at 5 °C, Figure S13 in Supporting Information, section H. Complementary ^1^H NMR studies, Figure S14 in Supporting Information, also show the formation and disappearance of different glycosylated and nonglycosylated intermediate species. The decay of glycosylated intermediates (and subsequent glucose release) follows the decrease of the aromatic signals identified as H-4 and H-2′6′ resonance signals from M3G dimers, suggesting that they represent the same compound. Moreover, the release of glucose appears to be slower than the formation of the dimers.

The gradual appearance of two new sets of ^1^H NMR signals (in different ratios) suggests the existence of two different M3G dimers, as also detected by MS (Scheme 5), which were tentatively assigned to the 4,8 and 4,6- A-type dimer using 1D and 2D NMR experiments, Figure S14 in the Supporting Information.

Bearing all this, it seems that M3G degradation at the transition pHs follows two different pathways; (1) condensation, followed by glucose hydrolysis, and (2) autoassociation (may be the first step) triggering condensation reactions. The formation of the dimer and respective oligomers in M3G increases dramatically along with the AC concentration.

Identical HPLC experiments were carried out for M3,5diG; see section I Supporting Information. In contrast to M3G, no clear evidence for the formation of colored dimers and oligomers was achieved. Moreover, the degradation rate of M3,5diG is much more slower than M3G, and after 7 days, ca. 33% of the flavylium cation is recovered in contrast with ca. 21% in the case of M3G after 1 day. The slower degradation rates of M3,5diG in comparison to M3G are also observed in the acidic medium. After standing M3G for 14 days at pH = 4, its disappearance is ca. 4%, while that of 3,5diG in identical conditions is <1%. This contrasts with the faster degradation rate of M3,5diG at higher pH values in comparison with M3G, as reported in Figure 9b,c (explained by the higher instability of B4^2–^).

In conclusion, a comprehensive study of the chemistry of ACs requires extension to the basic medium. The holistic approach presented in this work allowed the disclosure of a pH range (transition pHs), that includes the physiologic pH, in the Frontier between the acidic and basic paradigms, where hydration and OH^–^ nucleophilic addition are very slow. In this pH range, anionic quinoidal bases are the main reversible species, and complex degradation processes control their disappearance.

Detailed kinetic studies in basic conditions above the transition pHs allow for concluding that OH^–^ nucleophilic addition is the rate controlling step toward the equilibrium, and in contrast to acidic medium, the *cis*–*trans* isomerization is very fast. Despite the rate-limiting step toward the reversible equilibrium being common to malvidin mono and diglucoside, the latter presents a pre-equilibrium involving the anionic quinoidal base and a new transient species resulting from the OH^–^ attack in position 4, leading to the B4^2–^ species identified by ^1^H NMR.

The identity and concentrations of quinoidal bases, hemiketals, and *cis*-chalcones are obtained for the whole pH range from reverse pH jumps (to give flavylium cation at pH ≤ 1) monitored by stopped-flow, while *trans*-chalcones require standard spectrophotometry. The rate of the flavylium cation appearance in these experiments constitutes a kinetic signature that allows for the precise identification of the species and relative concentrations from the amplitude of the respective traces. Moreover, kinetic signatures are also useful to detect the existence of other species not observed in acidic pHs, as was the case of B4^2–^ for the diglucoside in the basic medium. No spectral evidence, from reverse pH jumps monitored by stopped flow experiments, for hemiketal anionic forms at the equilibrium was obtained.

## Supporting Information Available

Determination of the mole fraction distribution of all species by stopped flow, calculation of the acidity constants of the quinoidal bases, technical details in stopped flow measurements, identification of the kinetic reservoir as B4^2–^ in M3,5diG, computational details, and complementary studies of the M3G and M3,5diG degradation by NMR and HPLC techniques (PDF)
