# Supplementary material for: Comparing the Chemistry of Malvidin-3-O-glucoside and Malvidin-3,5-O-diglucoside Networks: A Holistic Approach to the Acidic and Basic Paradigms with Implications in Biological Studies
Source: J Agric Food Chem. 2024 Mar 23;72(13):7497–510. doi: 10.1021/acs.jafc.4c00552 (PMC10995998; doi:10.1021/acs.jafc.4c00552)
Supplement: Supplementary file 1 — jf4c00552_si_001.pdf [file jf4c00552_si_001.pdf]

SUPPLEMENTARY MATERIAL

Comparing the Chemistry of Malvidin-*3-O*-Glucoside and Malvidin-*3,5- O*-Diglucoside Networks: A Holistic Approach to the Acidic and Basic Paradigms with Implications in Biological Studies.

André Seco,[a#] Ana Rita Pereira,[b#] Ambrósio Camuenho,[a] Joana Oliveira,[b*] Ricardo Dias,[b] Natércia F. Brás,[b] Nuno Basílio,[a*] A. Jorge Parola,[a] João C. Lima,[a] Victor de Freitas,[b] and Fernando Pina[a*]

[a] LAQV – REQUIMTE, Departamento de Química, Faculdade de Ciências e Tecnologia, Universidade Nova de Lisboa, 2829-516 Caparica, Portugal, *E mail: fp@fct.unl.pt; nuno.basilio@fct.unl.pt

[b] LAQV – REQUIMTE, Departamento de Química e Bioquímica, Faculdade de Ciências, Universidade do Porto, Rua do Campo Alegre, 687, 4169-007 Porto, Portugal, jsoliveira@fc.up.pt

# These authors contributed equally to this work

Note 1 S2

A. Kinetic signatures and determination of the mole fractions of all species in two dimensions, pH and time S2

B. Calculation of the acidity constants of the quinoidal bases S4

C. Details in stopped flow measurements S5

D. Identification of the kinetic reservoir as $B4^{2-}$ in M3,5diG S5

E. Computational studies S7

F. Kinetics of M3G and M3,5diG S9

G. NMR and HPLC complementary studies S10

H. Studies of the M3G degradation monitored by HPLC and 1H NMR at low 5ºC S13

I- HPLC of M3,5diG S16

Note 1

Eq. (1) is valid when considering the equilibrium with the mono-anionic species and lack of B^- as observed in the reverse pH jumps monitored by stopped flow. However, the pH domain of Cc^- have a superposition with the pH range where anionic quinoidal bases are the only reversible species and thus is not reached. Moreover, representation of eq.1 versus pH using hypothetic values $7 < pK_{Cc/Cc^-} < 9$ is coincident with eq. 2 of the main text in less than 1%, justifying its use.

$$k_{hydration} = \frac{[H^+]^2}{[H^+]^2 + K_a[H^+] + K_a K_{A/A^-}} k_h + \frac{[H^+]}{(1+K_t)[H^+] + K_t K_{Cc/Cc^-}} k_{-h}[H^+] \quad (1)$$

A. Kinetic signatures and determination of the mole fractions of all species in two dimensions, pH and time

Reverse pH jumps from equilibrated or pseudo-equilibrated solutions at higher pH values back to flavylium cation pH≤1, monitored by stopped-flow, allow for the definition of the kinetic signatures of hemiketal and *cis*-chalcone. The amplitude at t=0 corresponds to the fraction of quinoidal bases. The kinetic signature of *trans*-chalcone requires the use of a standard spectrophotometer.

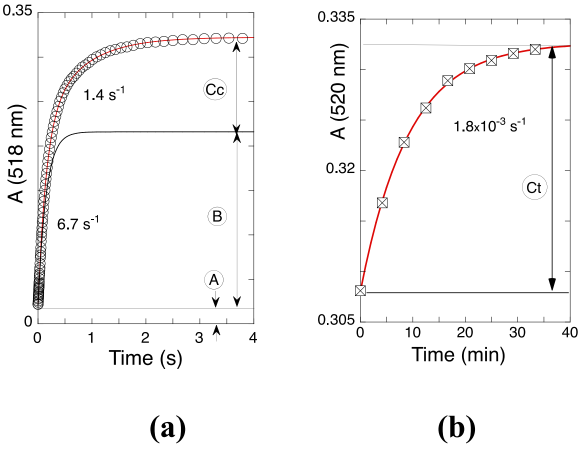

Figure S1. (a) Traces of a reverse pH jump of M3G from pH=5.7 to pH=0.9; **(b)** the same followed by a standard spectrophotometer. Estimated error 10%.

A reverse pH jump of M3G from pH=5.1 to pH=0.9 is shown in Fig.S1. The kinetic signature for this concentration of acid is 6.7 s^{-1} and 1.4 s^{-1} respectively for the conversion of hemiketal and *cis*-chalcone in flavylium cation. Another reverse pH jump from the same initial pH was

monitored by a standard spectrophotometer. The initial amplitude corresponds to all quinoidal base, hemiketal and *cis*-chalcone present at pH=5.7 prior to the reverse pH jump and the amplitude of the trace the fraction of *trans*-chalcone and the kinetic signature is 1.8x10^{-3} s^{-1}. This procedure could be extended to all pH range and could give the mole fraction of all species in two dimensions pH and time (carrying out the reverse pH jumps for several times).

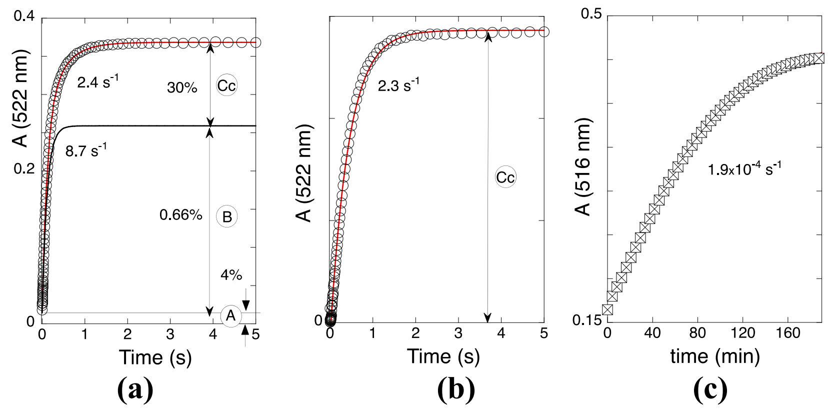

Figure S2. (a) Reverse pH jumps from pseudo-equilibrated solutions of M3,5diG at pH=4.2 to [HCl]=0.5 M, monitored by stopped-flow **(b)** the same from pH=12.8; **(c)** reverse pH jump from equilibrated solutions at pH=12.8 monitored by a standard spectrophotometer. Estimated error 10%.

The traces of the reverse pH jump from a solution of M3,5diG equilibrated at pH= 4.2 (Fig. S2a) and pH=12.8 (Fig. S2b) back to flavylium cation at [HCl]=0.5 M, are shown. The kinetic signature of the hemiketal in these conditions is 8.7 s^{-1} while *cis*-chalcone is 2.3 s^{-1}. It is worth of note the lack of hemiketal signature when the initial pH of the reverse pH jump is from pH=12.8, (Fig. S2b) indicating the lack of anionic hemiketal at this basic pH value. The kinetic signatures of *trans*-chalcone require the use of a standard spectrophotometer, Fig. S2c. Since at the equilibrium the fraction *trans*-chalcone is low the respective kinetic signature was taken from the equilibrium at pH=12.8.

A final remark regarding the accuracy of the calculation of pK_a from reverse measurements monitored by stopped-flow. It is difficult to calculate with precision the absorbance at the initial time. The best strategy to calculate the acid-base constants of the quinoidal bases is from the absorption spectra versus pH taken 10 ms after the Jump. A titration allows for obtaining not only pK_a but also pK_{A/A^-} and p$K_{A2-/A-}$, see below section B

B. Calculation of the acidity constants of the quinoidal bases

While it is not possible to follow the kinetics of the proton transfer by stopped-flow, the respective acidity constants can be calculated by collecting the absorption spectra 10 ms after the mixing.

M3G

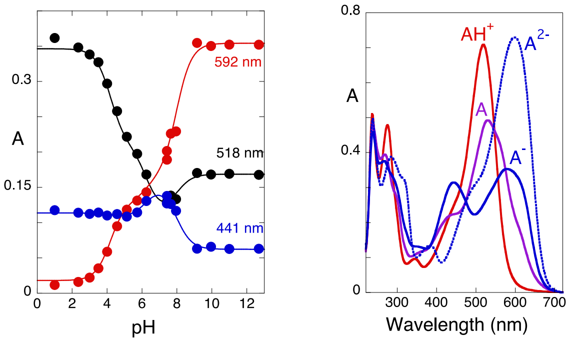

(a) **(b)**

M3,5GdiG

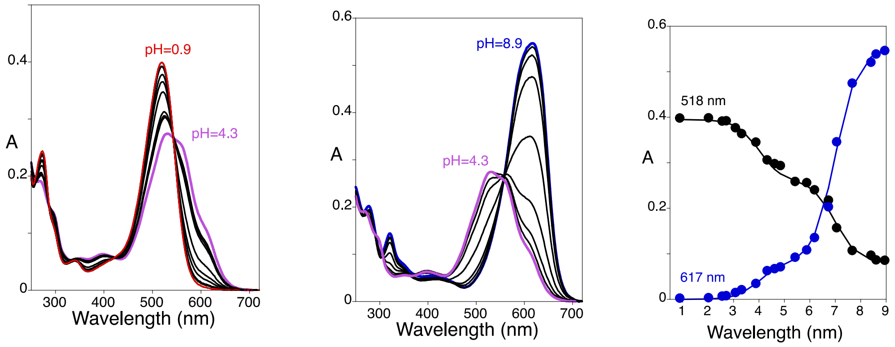

(c) **(d)** **(e)**

Figure S3. (a) Titration curves of M3G, 1.2×10^{-5} M, obtained from the absorption spectra taken 10 ms after a set of direct pH jumps monitored by stopped-flow. Fitting was achieved for pK_a=3.8, $pK_{A/A-}$=6.3 and $pK_{A-/A2-}$=8.1 are obtained[17,24], (b) deconvolution of the absorption spectra using the acid-base constants calculated in (a); (c) spectral variations of M3,5GdiG, 7.3×10^{-6} M, taken 10 ms after a direct pH jump monitored buy stopped-flow for 0.9<pH<4.3; (d) the same for 4.3<pH<8.9; (e) Titration curves at 518 nm and 617 nm. Fitting was achieved for pK_a=3.8 and $pK_{A/A-}$=7.0. Estimated error ±0.1.

In Fig.S3a the titration curves of M3G are presented as well as the deconvolution to calculate the absorption spectra of each species, Fig. S3b. Fitting was achieved for pK_a = 3.8, (to give quinoidal base), pK_{A/A^-} = 6.3 (to give anionic quinoidal base) and $pK_{A^-/A^{2-}}$ = 8.5 (to give di-anionic quinoidal base).[23] In the case of M3,5diG only two quinoidal bases can be formed, the neutral and the anionic and it is possible to separate the absorption spectra in two sets exhibiting isosbestic points, Fig.S3c and Fig.S3d, deconvolution is not necessary to have the absorption

spectra of these species. Fitting of Fig. S3e allows for the determination of pK_a=3.8 and pK_{A/A^-} =7.0, see Table S1.

Table S1. (1[st]step). Acid-base constants of (M3G) and [23] (M3,5diG) quinoidal bases. Estimated error 10%.

	pK_a	p$K_{A/A-}$	p$K_{A-/A2-}$
M3G	3.8	6.3	8.1
M3,5diG[(1)]	3.8	7.0	-

C. Details in stopped flow measurements

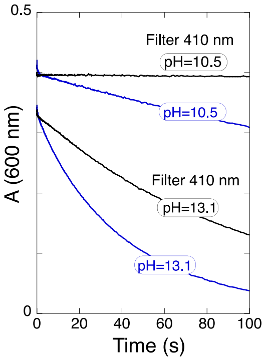

Figure S4. Stopped-flow traces after direct pH jumps of M3G with and without a 410 nm cutoff filter at two representative basic pH values.

Our stopped flow apparatus possesses a high intensity analyzing lamp and it is necessary to use short wavelength cutoff filters to prevent the photoinduced photochemical reactions that can occur from the *cis-trans* isomerization or tautomerization.[17,32]

Comparison of the stopped-flow traces with and without a cutoff filter (410 nm) at two representative pH values is shown in Fig. S4. In both cases (in the absence of the cutoff filter) it is clear the existence of a photo-induced reaction that consumes A^{2-}. However, the trace obtained at pH=10.5 in the presence of the 410 nm cutoff filter indicates that A^{2-} is not responsible for the photochemistry (because A^{2-} still absorbs light with this cutoff filter and its absorbance does not change). The very low intensity of the lamp of a standard spectrophotometer makes the interference of the photochemical reaction negligible

D. Identification of the kinetic reservoir as $B4^{2-}$ in M3,5diG

Figure S5, sumarizes two pH jumps, monitored by UV-Vis by means of a standard spectrophotometer, carried out at a concentration also suitable for the NMR experiments ($2x10^{-4}$ M) and 5º C. Considering that the acidity constants between AH^+ and A^- is pK=7 (Table 1

main text) and between A^- and $B4^{2-}$ is pK=10.7, a direct pH jump to pH=8.7 would convert almost 100% of flavylium cation in anionic quinoidal base, without significant formation of $B4^{2-}$, Fig. S5 dashed blue line. Conversely, after a direct pH jump to pH=11.9, the equilibrium between A^- to form $B4^{2-}$ is established (according to eq.5 at room temperature with a lifetime of 28 s), full red line in Fig. S5. In the experiments at 5ºC, we wait 15 min to assure that this equilibrium was reached.

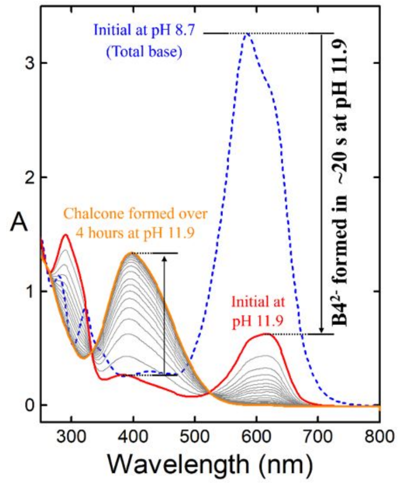

Figure S5. Spectral variations of M3,5diG (2.0x10^{-4} M, 5 °C) after a direct pH jump from pH=1 to pH=11.9 (full lines) and the first spectrum after a pH jump from pH=1 to pH=8.7 with the same concentration (dashed blue line).

The ratio of the absorbances ($A_{600}^{11.9}$ = 0.60 vs $A_{600}^{8.7}$ = 3.05) indicates that *ca* 80% of M3G was converted in $B4^{2-}$. In these conditions a relatively high concentration of this transient reservoir was formed.

Knowing this behavior we replicated the experiment monitoring the evolution of the system by ^{1}H NMR spectroscopy, using 10% D_2O to allow for the locking of the magnetic field but otherwise in the same conditions, including the same concentrations of anthocyanin and buffer. With suppression of the water signal (using the NOESY 1D pulse sequence) we were able to obtain a sequence of clear spectra over the course of *ca*. 24 hours, Fig. S6. In this experiment it is clear that there is a set of signals that is present at its highest intensity in the beginning and immediately starts to decrease in intensity. They can no longer be observed approximately after 2 hours. Also there is a set of signals that were attributed to the chalcones since they are increasing with time and another set that remain constant throughout the experiment, some of which we know to belong to the buffers used.

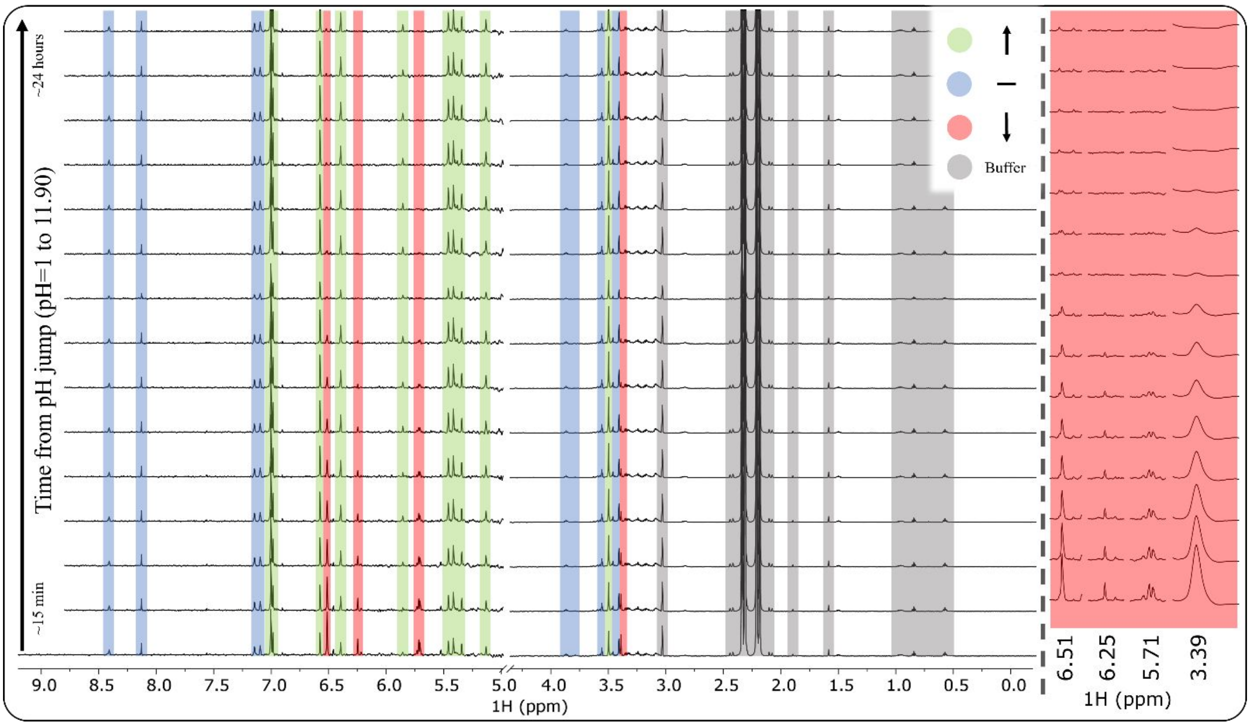

Figure S6. ^{1}H NMR consecutive spectra of M3,5diG (2.0x10^{-4} M) after a direct pH jump from pH=1 to pH=11.9 over *ca.* 24 hours. Highlighted in red are the signals whose intensity decreases until they can no longer be observed, in green the ones whose intensity increases over time, in blue and grey, the ones that remain practically unchanged over time, with the ones in grey corresponding to the signals of the buffers used to adjust the pH. The rightmost part of the image shows a zoom of the signals whose intensity decreases, highlighted in red. The irradiated water signal (4.66 ppm) was omitted for clarity.

Focusing our attention on the set of signals that decreases in intensity, the respective integrals were plotted against time and mono-exponential decays with an average kinetic constant of 4.3×10^{-4} s^{-1} were obtained, which compares well with the one determined for the formation of the chalcone (5.2×10^{-4} s^{-1}), taken from the data in Fig, S5, thus supporting our hypothesis that we are correctly identifying the signals of the kinetic reservoir species.

In order to isolate this set of signals, the last spectrum (after *ca.* 24 hours) was subtracted from the first one and the resulting difference spectrum, Fig. 7 main text, clearly shows the set of signals belonging to the transient species in agreement with our hypothesis of it being be $B4^{2-}$ due to their multiplicity and integration.

E. Computational studies

The Maestro software[33] was used to do the conformational search of all conformers of both malvidin-3-*O*-glucoside (M3G) and malvidin-3,5-*O*-diglucoside (M3,5diG) molecules. All geometries (292 and 327 of M3G and M3,5diG, respectively) were optimized using the hybrid B3LYP[34] density functional associated with the empirical dispersion D3 correction of Grimme

et al.,[35] in combination with the 6-31+G(d,p) basis set.[36] The aqueous solvent effects were accounted for by the SMD solvation model.[37] These calculations were performed by using the Gaussian 09 program package.[38] Harmonic vibrational frequency calculations were performed at the same level of theory to obtain the thermochemical corrections to the enthalpic and free energies at 298.15 K. The charge distribution was analyzed by applying the NBO formalism.[39]

Table S2. Charge distribution (Natural Bond Orbital (NBO) analysis) of some atoms as well as the HOMO/LUMO energy data at the SMD(water)/B3LYP-D3/6-31+G(d,p) level of theory, for the geometries with the lowest total energy of each compound. Charge differences > 0.1 are highlight at bold.

	atom	M3G (A^{2-})	M3,5diG (A^{-})	charge difference (M3,5diG – M3G)
AC moiety	O	-0.471	-0.467	+0.004
	C2	0.374	0.400	+0.026
	C3	0.189	0.234	+0.045
	O3	-0.594	-0.566	+0.028
	C4	-0.160	-0.185	-0.025
	H4	0.283	0.283	-0.000
	C4b	-0.188	-0.214	-0.026
	C5	0.399	0.336	-0.063
	O5	-0.868	-0.594	**+0.274**
	C6	-0.477	-0.350	**+0.127**
	H6	0.240	0.263	+0.023
	C7	0.406	0.395	-0.011
	O7	-0.853	-0.840	+0.013
	C8	-0.424	-0.409	+0.015
	H8	0.260	0.267	+0.007
	C8b	0.355	0.394	+0.039
	total	-1.529	-1.053	**+0.476**
B ring	C1'	-0.156	-0.169	-0.013
	C2'	-0.306	-0.287	+0.019
	H-C2’	0.273	0.274	+0.001
	C3'	0.230	0.227	-0.003
	O-C3'	-0.573	-0.566	+0.007
	C3'-O-C	-0.345	-0.348	-0.003
	C3'-O-C-H1	0.223	0.223	0.000
	C3'-O-C-H2	0.222	0.223	0.001
	C3'-O-C-H3	0.245	0.245	0.000
	C4'	0.318	0.352	+0.034
	O4'	-0.853	-0.800	+0.053
	C5'	0.188	0.249	+0.061
	O-C5'	-0.618	-0.568	+0.050
	C5'-O-C	-0.337	-0.329	+0.008
	C5'-O-C-H1	0.218	0.226	+0.008
	C5'-O-C-H2	0.237	0.248	+0.011
	C5'-O-C-H3	0.22	0.226	+0.006
	C6’	-0.260	-0.297	-0.037
	H-C6’	0.270	0.271	+0.001
	total	-1.074	-0.871	**+0.203**
MO	HOMO	-0.18001	-0.18103	-0.001
	LUMO	-0.08013	-0.09312	-0.013

F. Kinetics of M3G and M3,5diG

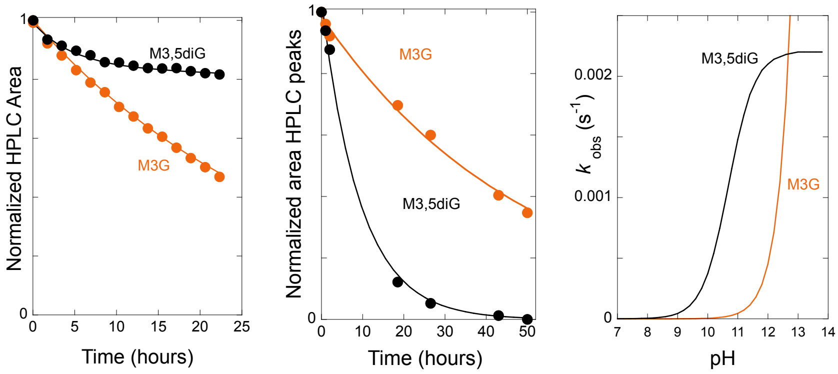

Figure S7. (a) Normalized area of the HPLC peaks of M3G (orange points) and M3,5diG (black points) at the physiologic pH, after 1 day. Fitting was achieved with a mono-exponential with rate constant 9.1x10^{-6} s^{-1} for M3G and biexponential with rate constants 8.9x10^{-5} s^{-1} (but only 12% of the total amplitude) and *circa* 9.8x10^{-7} s^{-1} for M3,5diG; **(b)** The same of (a) for pH=9.2, with rate constants 5.5x10^{-6} s^{-1} for M3G and 3x10^{-5} s^{-1} for M3,5diG; **(c)** Rates of the two compounds toward the equilibrium versus pH in basic medium.

The normalized area of the HPLC peaks of M3G and M3,5diG versus time after a direct pH jump to the physiological pH is presented in Fig. S7a. The fitting was achieved with a mono-exponential with rate constant 9.1x10^{-6} s^{-1} for M3G and 9.8x10^{-7} s^{-1} for M3,5diG, indicating that the monoglucoside degrades much faster.

In Fig. S7b an analog experiment was performed at pH=9. It is clear the inversion of stability of the two compounds in basic medium. Fitting was achieved with mono-exponentials 5.6x10^{-6} s^{-1} and 3x10^{-5} s^{-1} respectively for M3G and M3,5diG.

When comparing the rate of M3G disappearance at the physiologic pH in Fig. S7a, (9.1x10^{-6} s^{-1}) with the rates reported in Fig.8b, (4.0x10^{-6} s^{-1}) of the main text it is clear that the rates obtained from HPLC are higher than those monitored by UV-Vis spectrophotometry, which can be explained by the contribution of the degradation products exhibiting absorption spectra in the visible region. Conversely, the rates of M3,5diG are similar in Fig. S7a and Fig. 8c of the main text.

When the HPLC areas of the lyophilized sample of M3G (see experimental section for more details) after 2 days are integrated the observed rate constant (Fig. S8) of the colored forms is similar to the rate constant calculated by means of eq.(6) of the main text. This result can be explained if the mean averaged value of the mole absorption coefficients (ε Beer law) of the dimer and oligomers is similar to the one of the flavylium cation.

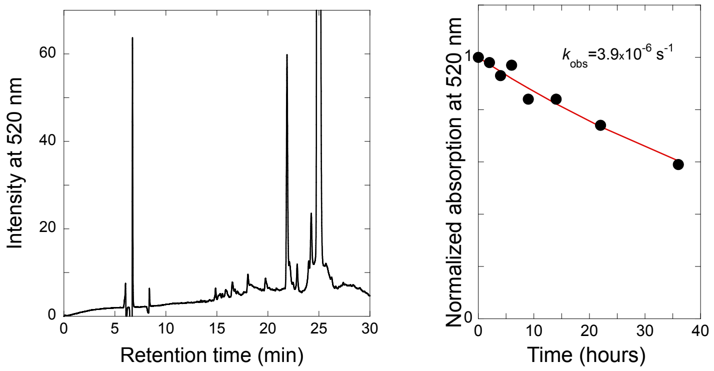

(a) **(b)**

Figure S8. (a) HPLC profile of the lyophilized sample of (3.3x10^{-5} M) after 2 days at pH=8.1; **(b)** Normalized areas upon integration of all peaks of (a) as a function of time.

G. NMR and HPLC-DAD complementary studies.

The degradation of M3G at 1x10^{-4} M at pH=8.1 was also monitored by ^{1}H NMR spectroscopy for 7 consecutive days. At time 0 (T_0), three main proton signals were detected in the aromatic region, Fig. S9. These were identified as corresponding to the H-4 (δ = 8.28 ppm), H-2',6' (δ = 7.63), and H-6 (δ = 6.22) of M3G ring C, B, and A, respectively. Over time, all proton signals were found to gradually become less intense, particularly within the first 72 hours, whose relative integrals reached 10% of their initial value, Fig. S9. For H-6,8, the behaviour was somewhat different from the previous ones due to a hydrogen-deuterium (H/D) exchange effect, whose kinetics was faster than their chemical decay. Of the four main by-products expected through the conventional degradation pathway, only syringic acid and glucose were detected by 1D and 2D NMR, respectively. Though non-quantifiable by ^{1}H NMR (when following the anomeric H-1 signal), glucose was found to be completely released from M3G at the end of 7 days, as confirmed by ^{1}H-^{1}H COSY spectroscopy using D-glucose as an external standard, Fig. S10. Due to the limited ^{1}H sensitivity of NMR spectroscopy at such a low M3G concentration, no reaction intermediaries or oligomerization phenomena could be detected, nor could other 2D approaches like DOSY (Table S3) or T_1/T_2 measurements (Fig. S12) be employed in a workable timeframe.

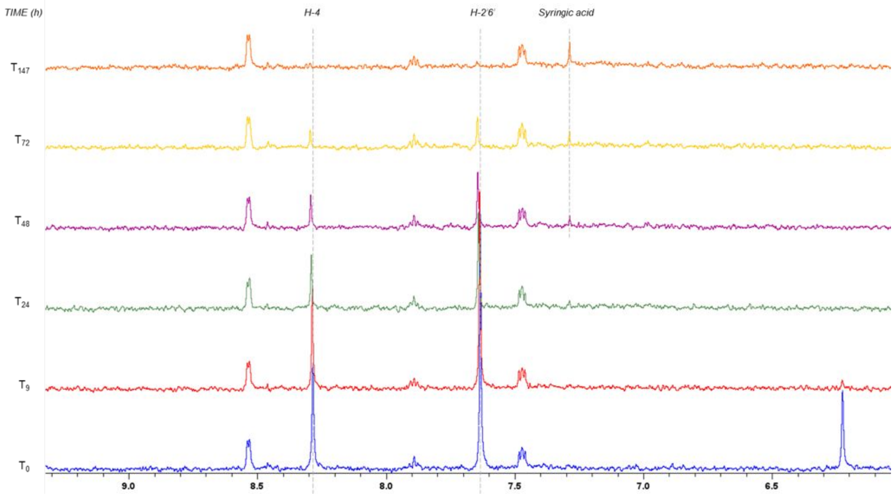

Figure S9. ^{1}H NMR spectra in the aromatic region of M3G at $1x10^{-4}$ M taken at different time points. Spectra were acquired at 25 °C. pH=8.1

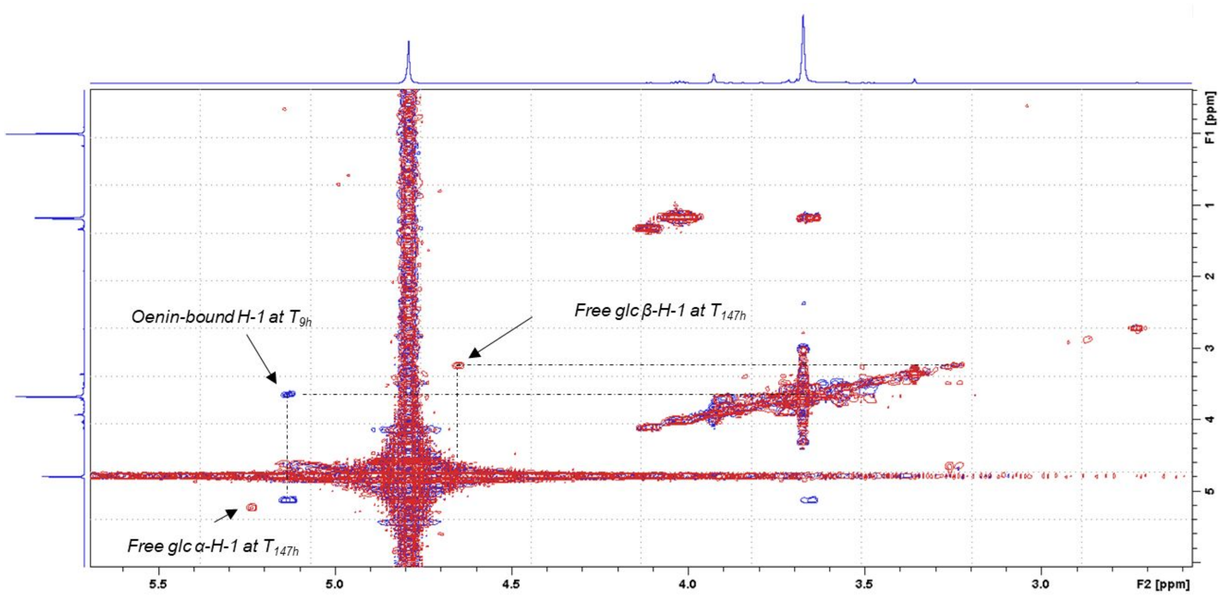

Figure S10 - Superimposition of M3G ^1H-^1H correlation spectra at T_{0h} and T_{147h} highlighting the hydrolysis of glucose molecules from the parental structure into the solvent media. In solution, glucose can exist in two anomeric forms: α-D-glucose and ß-D-glucose.

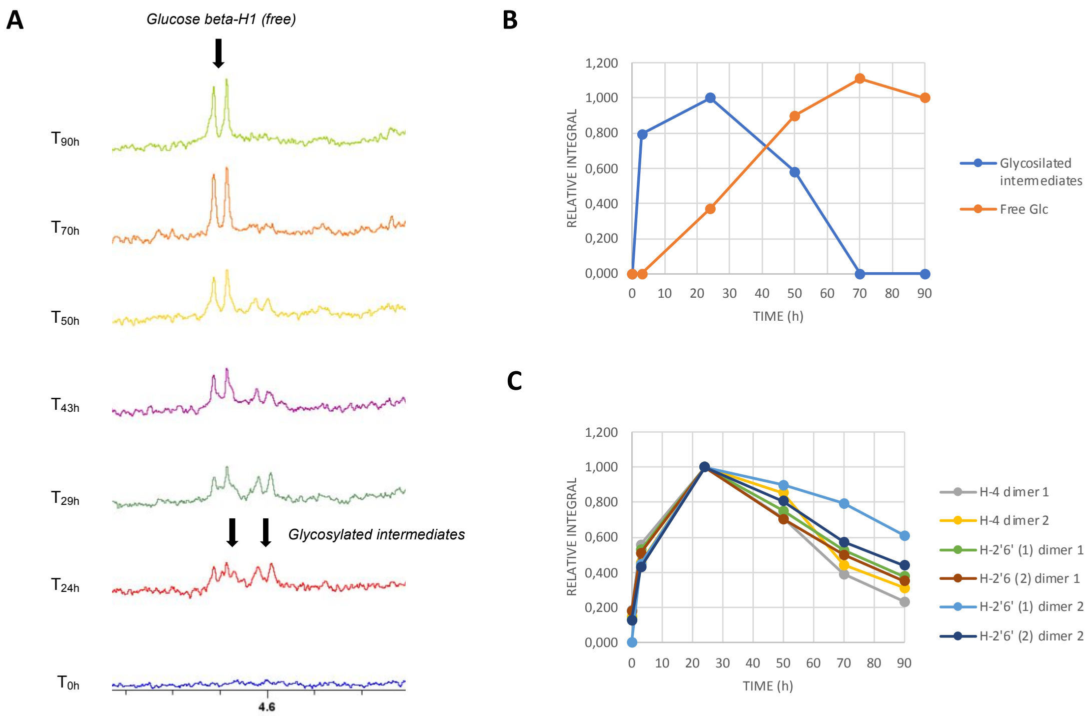

Figure S11. a) Zoom-in of M3G NMR spectra (acquired at 5 °C) in the anomeric 1H region of D-glucose, where the appearance and disappearance of glycosylated intermediates was detected **(b).** The latter were found to correspond to type-A C4-C6 and C4-C8 M3G dimers whose kinetic decay accompanies the ones observed for their corresponding H-4 and H-2’6’ aromatic signals (c).

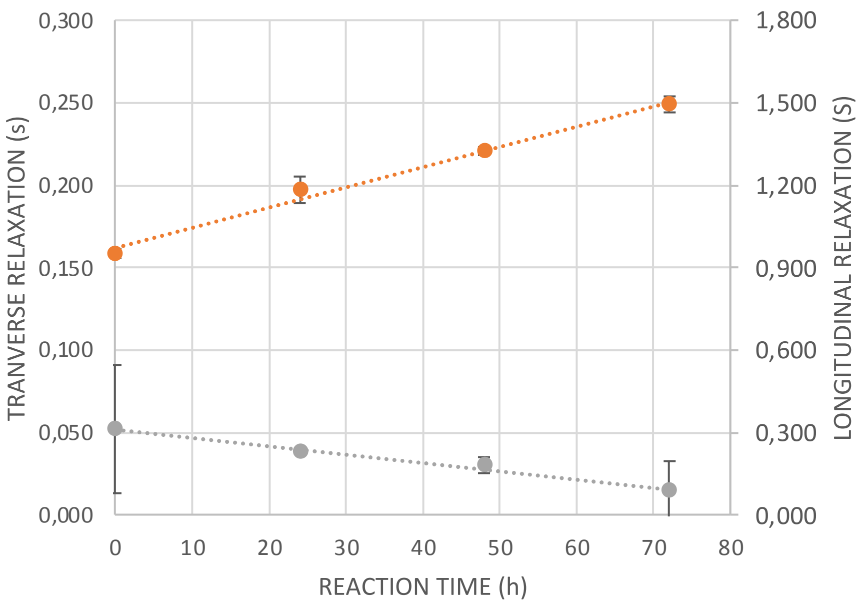

Figure S12. T1/T2 relaxation measurements (determined for M3G H-4) over time.

Table S3 - Diffusion coefficients determined at 5 and 25ºC for different protons signals during M3G degradation.

	5 °C		25 °C	
	**Chemical Shift (ppm)**	**Diffusion coefficient (m^2/s)**	**Chemical Shift (ppm)**	**Diffusion coefficient at (m^2/s)**
**TSP**	0.00	$3.98 \times 10^{-10} \pm 5.58 \times 10^{-12}$	0.00	$8.59 \times 10^{-10} \pm 6.17 \times 10^{-12}$
**M3G H-4**	8.04	$1.95 \times 10^{-10} \pm 3.69 \times 10^{-12}$	8.13	$3.54 \times 10^{-10} \pm 5.49 \times 10^{-12}$
**M3G H-2'6'**	7.33	$1.87 \times 10^{-10} \pm 3.42 \times 10^{-12}$	7.45	$4.30 \times 10^{-10} \pm 2.52 \times 10^{-12}$
**M3G dimer 1 (H-2'6')**	6.72	$1.74 \times 10^{-10} \pm 3.30 \times 10^{-12}$	---	---
**M3G dimer 2 (H-2'6')**	6.80	$1.85 \times 10^{-10} \pm 7.66 \times 10^{-12}$	---	---
**Conjugated glucose**	4.59	$1.50 \times 10^{-10} \pm 1.07 \times 10^{-11}$	---	---
**(D)-Glucose**	4.65 (ß)	$4.32 \times 10^{-10} \pm 4.62 \times 10^{-12}$	5.24 (α)	$8.26 \times 10^{-10} \pm 2.61 \times 10^{-11}$
**(D)-Glucose Standard**			5.24 (α)	$8.31 \times 10^{-10} \pm 3.55 \times 10^{-12}$

H. Studies of the M3G degradation monitored by HPLC-DAD and ^{1}H NMR at 5ºC

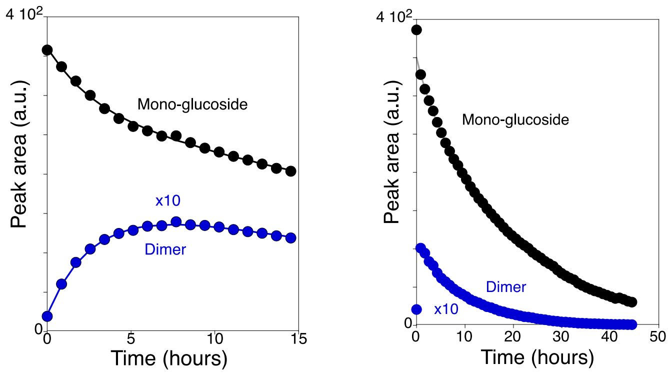

(a) **(b)**

Figure S13. (a) Degradation kinetic of pH=8.1, (1 mM) at 5ºC (black) and formation of dimer intermediate (blue); fitting of both curves can be achieved with a bi-exponential of rate constants k_1=1.0x10^{-4} s^{-1} and k_2= 6.3x10^{-6} s^{-1}; **(b)** the same of (a) at room temperature; neglecting the two first points the fitting can be achieved with a mono-exponential with rate constants for the monoglucoside and dimer respectively 1.5x10^{-5}s^{-1} and 3.0x10^{-5}s^{-1}.

The studies of the M3G degradation monitored by HPLC-DAD at low temperature are shown in Fig.S13. Fitting of the traces reported in Fig.S13a are achieved with a biexponential with the same rate constants k_1=1.0x10^{-4} s^{-1} and k_2= 6.3x10^{-6} s^{-1}. The first process can be associated to the disappearance of M3G responsible for the appearance of the dimer. Further conclusions regarding the second-rate constant in this experiment are limited by its low accuracy. The data at 5ºC permits to rationalize the results at room temperature, Fig. S13. The two first points are still those remaining from the dimer formation. The rest of the decay regards the degradation

of the monoglucoside, $1.5x10^{-5}s^{-1}$, and dimer, $3.0x10^{-5}s^{-1}$, both achieved with a mono-exponential.

The formation/turn-over of any potential intermediate was also followed by ^{1}H NMR, Fig. S14. Through analysis of the major spectral changes occurring in a 5-day window, and like was observed at 25 °C, Fig. 10c of the main text, several different intermediate species were found to gradually arise and decay over time, some glycosylated, Fig. S11, though in these conditions 1 week wasn't still enough to get a spectral pattern as crowded as the one obtained at 25 °C at T_0. The decay of glycosylated intermediates (and subsequent glucose release) is followed by a similar decrease of the aromatic signals identified as H-4 and H-2'6' resonance signals from M3G dimers. As such they should represent the same compound. Moreover, the release of glucose appears to be secondary to the formation of M3G oligomers (slow process at NMR timescale). In addition, dimers seem to be in equilibrium with larger species, leading to larger peak width at half height and broader glucose signals.

As expected, there is a temperature dependence of the M3G diffusion coefficient, Table S3. The signal(s) (*i.e.* moiety) identified before as the anomeric beta-H-1 of glucose from dimers have a lower diffusion coefficient than glucose when freed, and therefore a higher molecular weight. Of note, diffusion coefficients were determined at T_{24h} (where intermediate dimer signals reached their maximum intensity) except for D-glucose whose diffusion coefficient was calculated at t_{90h}. Also, M3G appears to be in fast exchange with heavier structural conformers (with shorter T2 values) characterized by a high degree of order (reflected by slow longitudinal (T1) relaxation) and these effects increase over time, Fig. S12.

A close inspection of Fig. S14b and knowing that two M3G dimers were detected by mass spectrometry in the positive ion mode (as described above), a tentative assignment of these intermediates was accomplished by 1D and 2D proton NMR. It can be observed in the ^{1}H NMR spectrum the appearance over time of two protons (in different ratios) corresponding to the protons H-4 of the terminal units of two M3G dimers. At 6.9-6.6 ppm, two sets of signals corresponding to protons H-2',6' were present at the B-ring of the terminal, and the extension units of each M3G dimer were also found to occur. According to these results, two possible structures could be assigned to the M3G dimer as presented in Scheme 5, the A and the B-type dimers. From the 2D analysis (COSY), it was possible to observe two correlations at around 2.6 ppm with protons at 3.3 ppm that should correspond to protons H-3 and H-4 from ring C of the flavene extension unit, Fig. S14, which indicates that the linkage between the two M3G units should correspond to an A-type linkage as previously described to occur in grapes and

wines. The presence of two isomers should correspond to C-4/C-8 (major isomer) and C-4/C-6 (minor isomer), Fig S14.

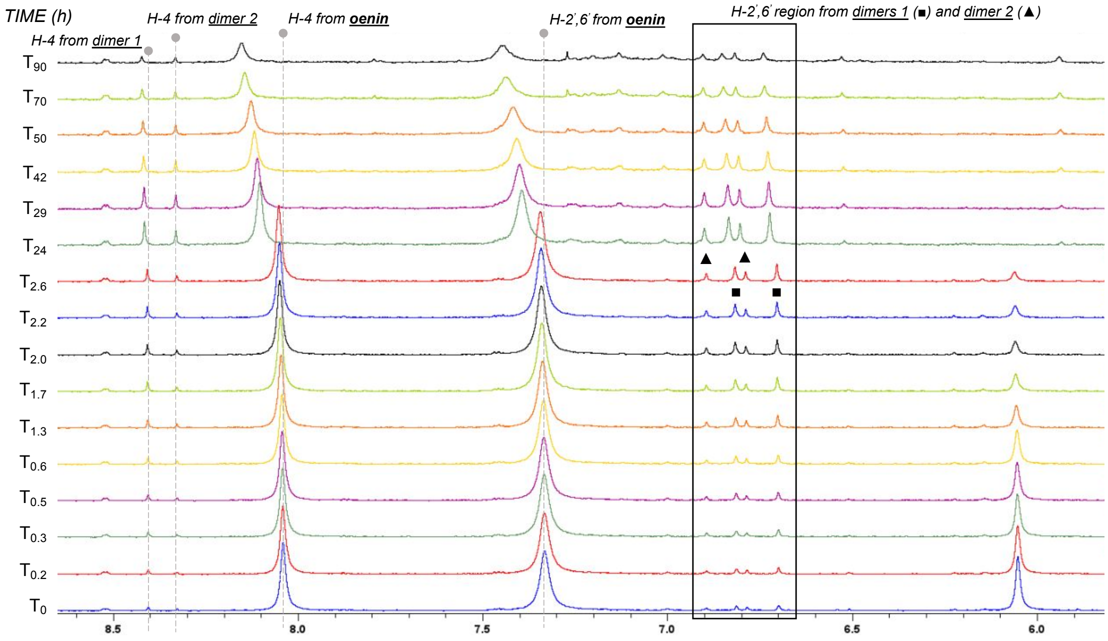

(a)

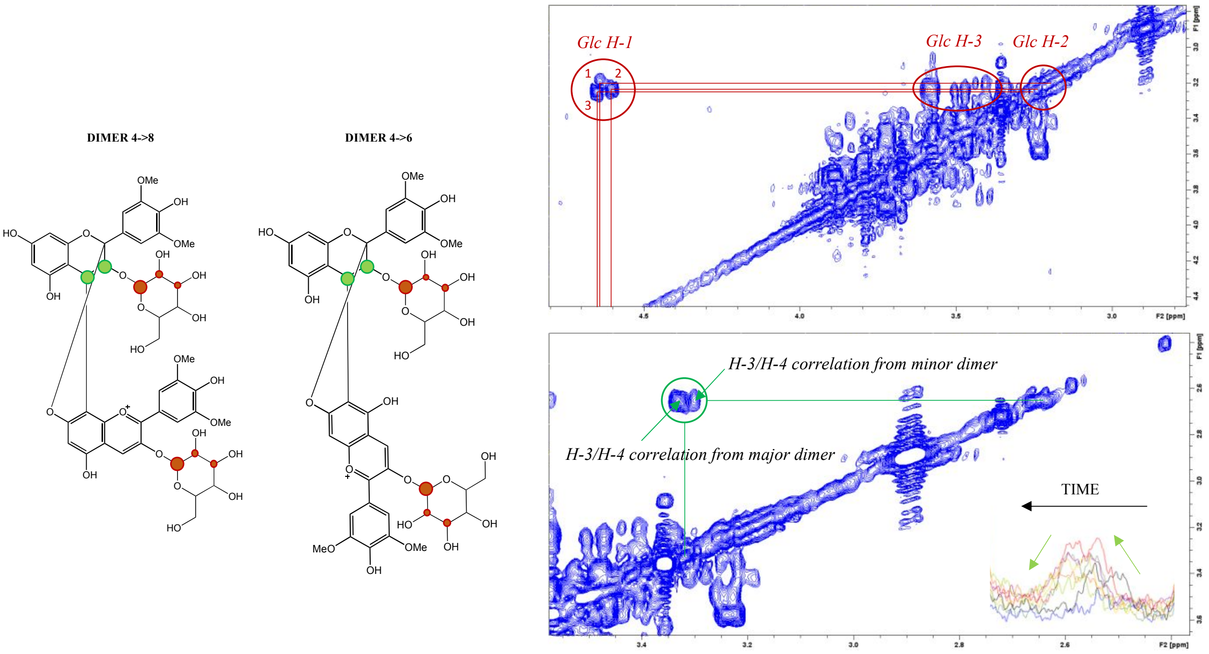

(b)

Figure S14. a) ^{1}H NMR spectra in the aromatic region of at 1x10^{-3} M taken at different time points. Spectra were acquired at 5 °C. b) COSY spectra showing H-3/H-4 correlation from ring C of the extension unit of dimers and correlations of anomeric proton H-1 and protons H-2 and H-3 from the glucose moiety. 1. Anomeric beta-H-1 from minor dimer (2 moieties); 2. Anomeric beta-H-1 from major dimer (2 moieties); 3. Anomeric beta-H-1 from glucose (by chemical shift referencing).

I. HPLC of M3,5diG

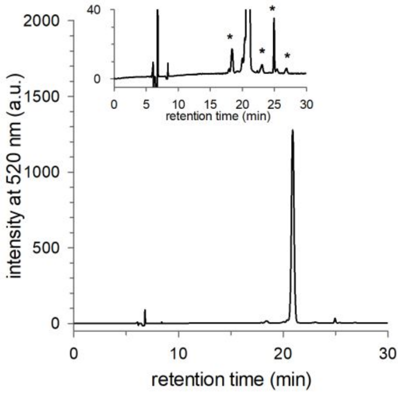

Figure S15. Control HPLC chromatogram*s* of M3,5diG stock solution at pH 1 ($3x10^{-4}$ M). Unidentified impurities are marked with an asterisk (*) in the inset.

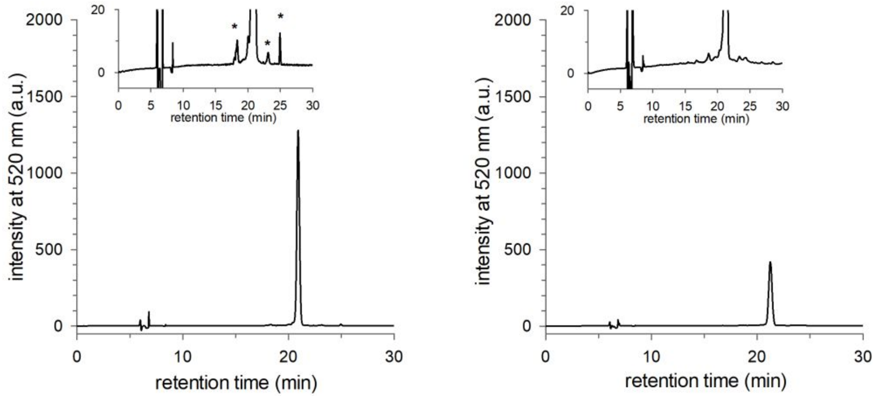

(a) **(b)**

Figure S16. HPLC chromatogram*s* of M3,5diG solution for the representative transition pH 7.4 ($3x10^{-4}$ M) **(a)** after 2 hours*;* **(b)** after 7 days. Unidentified impurities are marked with an asterisk (*) in the inset.
